# The Molecular Basis for Control of ETEC Enterotoxin Expression in Response to Environment and Host

**DOI:** 10.1371/journal.ppat.1004605

**Published:** 2015-01-08

**Authors:** James R. J. Haycocks, Prateek Sharma, Anne M. Stringer, Joseph T. Wade, David C. Grainger

**Affiliations:** 1 Institute of Microbiology and Infection, School of Biosciences, University of Birmingham, Edgbaston, Birmingham, United Kingdom; 2 Wadsworth Center, New York State Department of Health, Albany, New York, United States of America; 3 Department of Biomedical Sciences, School of Public Health, University at Albany, SUNY, Albany, New York, United States of America; National Institutes of Health, United States of America

## Abstract

Enterotoxigenic *Escherichia coli* (ETEC) cause severe diarrhoea in humans and neonatal farm animals. Annually, 380,000 human deaths, and multi-million dollar losses in the farming industry, can be attributed to ETEC infections. Illness results from the action of enterotoxins, which disrupt signalling pathways that manage water and electrolyte homeostasis in the mammalian gut. The resulting fluid loss is treated by oral rehydration. Hence, aqueous solutions of glucose and salt are ingested by the patient. Given the central role of enterotoxins in disease, we have characterised the regulatory trigger that controls toxin production. We show that, at the molecular level, the trigger is comprised of two gene regulatory proteins, CRP and H-NS. Strikingly, this renders toxin expression sensitive to both conditions encountered on host cell attachment and the components of oral rehydration therapy. For example, enterotoxin expression is induced by salt in an H-NS dependent manner. Furthermore, depending on the toxin gene, expression is activated or repressed by glucose. The precise sensitivity of the regulatory trigger to glucose differs because of variations in the regulatory setup for each toxin encoding gene.

## Author Summary

Diarrheagenic illness remains a major disease burden in the developing world. Enterotoxigenic *Escherichia coli* (ETEC) are the leading bacterial cause of such disease; hundreds of millions of cases occur every year. The severe watery diarrhoea associated with ETEC infections results from the action of enterotoxins. The toxins target human gut epithelial cells and trigger the loss of water and electrolytes into the gut lumen. Oral rehydration therapy can counteract this process. Hence, glucose and salt solutions promote rehydration of the patient. In this work we show that the gene regulatory mechanisms controlling toxin expression respond directly to sugar and salt. Furthermore, we describe a molecular mechanism to explain these effects. Hence, we provide a starting point for the optimisation of oral rehydration solutions to reduce toxin expression over the course of an ETEC infection.

## Introduction

ETEC are Gram negative bacteria that cause severe diarrhoea, known as non-*vibrio* cholera, in humans [1], [2]. First isolated in 1971, ETEC are responsible for 210 million infections annually, mostly in developing countries, leading to 380,000 deaths [3]. Disease results primarily from the action of two enterotoxins. The heat-labile toxin (LT) is similar in structure and function to cholera toxin [4], [5]. The heat-stable toxin (ST) mimics the human hormone guanylin [6]. Both toxins are secreted by ETEC during infection. Made up of two subunits, encoded by the *eltAB* operon, LT has the configuration AB_5_ [5], [7]. In the gut, LT binds to host cell GM1 gangliosides and is endocytosed [8], [9]. This triggers constitutive cAMP production in the affected cell [8]. The ST toxin, encoded by the *estA* gene, also interferes with cell signalling [6]. Hence, ST binds to the guanylate cyclase C receptor and stimulates overproduction of cGMP. The combined actions of LT and ST cause loss of H_2_O, and electrolytes, from epithelial cells into the gut lumen [4]. Oral Rehydration Therapy (ORT) is used to redress the resulting electrolyte imbalance and rehydrate the patient [10]. In its most simple form, ORT requires only an aqueous solution of glucose and salt. Hence, the availability of metabolites and cations are a central theme of ETEC mediated disease. The effect of ORT on human physiology is well understood: glucose and Na_2_^+^ are transported across the epithelial membrane, along with water, to promote rehydration [11]. Surprisingly, despite the existence of molecular mechanisms that allow bacteria to respond to these signals, the consequences for ETEC are unknown.

In *E. coli*, the transcriptional response to glucose is controlled by cAMP receptor protein (CRP) [12]. In the absence of glucose, intracellular cAMP levels increase and CRP binds DNA targets with the consensus sequence 5′-TGTGA-n_6_-TCACA-3′ [13]. Subsequently, gene expression is reprogrammed to make use of alternative carbon sources [14]. Note that the gene regulatory network managed by CRP includes many indirect pathways [14], [15]. Hence, CRP is also a pleiotropic regulator of transcription. Whilst indirect regulatory effects are difficult to characterise, genes that are directly controlled by CRP can be divided into distinct classes [12]. At Class II targets, CRP binds to a site overlapping the promoter -35 element and interacts directly with both the N-terminal and C-terminal domains of the RNA polymerase α subunit (αNTD and αCTD). At Class I targets, CRP binds further upstream and interacts only with αCTD. This interaction can be further stabilised by UP-elements, AT-rich DNA sequences, adjacent to the CRP site, that facilitate αCTD-DNA interactions [12]. At both classes of promoter, the various contacts enhance gene expression by stabilising the transcription initiation complex. Unsurprisingly, most genes regulated by CRP encode proteins involved in metabolism. However, in some bacteria, CRP has been co-opted as a virulence regulator [16].

The Histone-like Nucleoid Structuring (H-NS) factor is a component of bacterial nucleoprotein. Consequently, H-NS also influences gene expression on a global scale [17]. Briefly, H-NS targets sections of the genome with a low GC content [17]. Depending on H-NS conformation, the resulting nucleoprotein complexes can be filamentous or bridged in organisation [18]. Filamentous complexes favour gene regulation by excluding RNA polymerase, and transcriptional regulators, from their targets [19], [20]. Bridged complexes favour RNA polymerase trapping [21]. In all scenarios, it is thought that H-NS acts primarily to silence transcription [22]. The conformation of H-NS, and hence the way in which it modulates DNA topology, can be controlled by divalent cations. Consequently, H-NS mediated repression can be relieved by increased osmolarity [23]. Like CRP, H-NS has been incorporated into the virulence gene regulatory networks of many bacteria [17].

In this work we define the molecular trigger that controls toxin expression in ETEC. We show that CRP and H-NS are key regulatory factors. Strikingly, this allows ETEC to integrate extracellular signals of osmolarity and metabolism to control toxin production. Hence, we propose that ETEC toxicity responds directly to osmo-metabolic flux. Interestingly, the precise regulatory settings are different for each toxin encoding gene. The differences result from i) varying promoter configurations and ii) competition between CRP and H-NS for overlapping DNA targets. This is significant since fluctuations in osmolarity, and changes in the availability of metabolites, are central to ETEC infection and its treatment.

## Results

### Binding of CRP and H-NS across the ETEC H10407 genome

**Figure 1:**
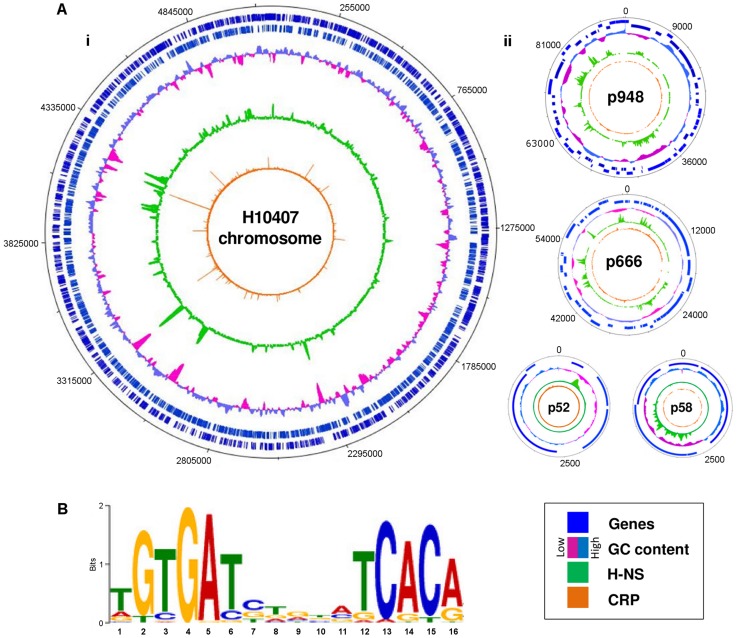
Distribution of CRP and H-NS across the ETEC H10407 genome. A) The panel shows maps of the ETEC H10407 chromosome (i) and associated plasmids (ii). In each plot, tracks 1 and 2 (blue lines) show the position of genes, track 3 (purple and cyan graph) is a plot of DNA GC content, track 4 (green) is the H-NS binding profile and track 5 (orange) is the CRP binding profile. B) A DNA sequence motif generated by aligning regions of the ETEC H10407 chromosome bound by CRP.

The prototypical ETEC strain H10407 reproducibly elicits diarrhoea in human volunteers and has a well-defined genome that shares 3,766 genes with *E. coli* K-12 [1]. Pathogenicity arises from 599 ancillary genes encoded by 25 discrete chromosomal loci and 4 plasmids. The plasmids, named p948, p666, p58 and p52, encode the enterotoxins. Derivatives of the *estA* gene are found on plasmids p666 (e*stA1*) and p948 (e*stA2*). A single copy of the *eltAB* operon is encoded by plasmid p666. We used Chromatin Immunoprecipitation (ChIP) coupled with next-generation DNA sequencing (ChIP-seq) to map CRP and H-NS targets across the ETEC H10407 genome. The binding profiles are shown in Fig. 1A. In each plot genes are illustrated by blue lines (tracks 1 and 2), DNA G/C content by a cyan and pink graph (track 3), H-NS binding is in green (track 4) and CRP binding is shown in orange (track 5). As expected, H-NS binding is inversely correlated with DNA G/C content (compare tracks 3 and 4). Similarly, CRP binding occurs in expected locations; 96% of the CRP binding sites are associated with the DNA logo shown in Fig. 1B (i.e. the known CRP consensus sequence (13–15)). We identified a total of 111 high-confidence CRP targets (Table 1). Of these targets 93% were present in the genome sequences of both ETEC H10407 and *E. coli* K-12. The most common location for CRP sites was in intergenic regions (66% of targets) whilst a smaller number of targets were found within genes (34%). Consistent with expectations, CRP sites were most frequently located ∼40.5 bp, or ∼92.5 bp, upstream of experimentally determined transcription start sites (TSSs). Surprisingly, CRP binding was restricted to the ETEC chromosome (Fig. 1Ai). Conversely, H-NS bound to chromosomal and plasmid loci (Fig. 1Ai), including all toxin encoding genes (Fig. 1Aii).

**Table 1:** High-confidence CRP binding sites on the ETEC H10407 chromosome identified by ChIP-seq.

Peak Centre^a^	Binding Site(s)^b^	Gene(s)^c^	K-12 Homologues^d^
45284	**TGTGA**TTGGTA**TCACA**	*ETEC_0040*	*caiT*
92014	A**GTGA**TGGATG**TCAC**G	(*ETEC_0078*)	(*cra*)
176905	A**G**C**G**TTCCACG**TCACA**	*(ETEC_0150)*	*(hemL)*
408885	**TGTGA**TCTCTC**TC**G**CA**	*ETEC_0385*/*ETEC_0386*	*yahN*/*yahO*
461874	**TGTG**CGCAAGA**TCACA**	*ETEC_0434*	*ddlA*
463095	TT**TG**CGCGAGG**TCACA**	*(ETEC_0436)*	*(phoA)*
468009	A**G**G**GA**TCTGCG**TCACA**	*ETEC_0443*	*aroM*
492973	ATC**GA**TTGCGT**TCAC**G	*ETEC_0464*	***tsx***
540805	**TGTGA**TCTTTA**TCACA**	*ETEC_0511*	*maa*
574230	GA**TGA**CGACGA**TCACA**	*(ETEC_0538)*	*(ybaT)*
683187	A**GTGA**TCGAGT**T**A**ACA**	*ETEC_0628*	***cstA***
697540	A**GTGA**TTTGCG**TCACA**	*ETEC_0639*	*rnk*
739223	C**GT**T**A**CCCTTG**TC**G**CA**	*ETEC_0680*	*rihA*
941002	**TGTGA**TGAGTA**TCAC**G	*ETEC_0869*	*ybiJ*
958866	**TGTGT**ACGAAA**TCACA**	*ETEC_0886/ETEC_0887*	***ybiS/ybiT***
1128472	n.d.	(*ETEC_1030*)	(*yccS*)
1205350	A**GTGA**TGTAGA**TCACA**	*ETEC_1101*	*ycgZ*
	**TG**A**GA**TCGAGCA**CACA**		
1263558	**T**T**TGA**CGGCTA**TCAC**G	*ETEC_1166*	***ptsG***
1274886	**TGTGA**TCTGGA**TCACA**	*ETEC_1176*/*ETEC_1177*	***ycfQ***/***bhsA***
1301786	GA**TGA**TCCGCA**TCACA**	(*ETEC_1206*)/*ETEC_1207*	ETEC-specific/ETEC-specific
1348166	AT**TGA**ACAGGA**TCACA**	*(ETEC_1259)/ETEC_1260*	*(rluE)/icd*
1376374	G**GTGA**GCTGGC**TCACA**	*ETEC_1292*/*ETEC_1293*	***ycgB***/***dadA***
1388620	A**GTGA**GCCAGT**TAACA**	*(ETEC_1303)*	*(dhal)*
1541732	CG**TGA**ACCGGG**TCACA**	*ETEC_1443/ETEC_1444*	*ycjZ/mppA*
1567885	GTTA**A**GTAAAA**TCACA**	*ETEC_1462/ETEC_1463*	***paaZ/paaA***
1701402	**TGTGA**TGGATG**TCACT**	*ETEC_1568*	*ydeN*
1767726	**TGTGA**TTAACAG**CACA**	*ETEC_1628*	***mlc***
1777143	**TGTGA**TCTAGCG**C**CA**A**	*ETEC_1637*	*pntA*
1811426	CG**TGA**TCAAGA**TCACG**	*(ETEC_1668A)*	(ETEC specific)
1859265	AT**TGA**GCGGGA**TCACA**	*(ETEC_1713)*	*(sufS)*
1887513	A**GTGA**TGCGCA**TCAC**G	*ETEC_1737*	*aroH*
	**TG**C**GA**GGTGTG**TCACA**		
2126754	**TGTG**GCGTGCA**TCACA**	n.a.	n.a.
2201816	G**GTGA**CGCGCG**TCACA**	*ETEC_2057*	*yedP*
2210222	C**GTGA**TCTCGCG**CACA**	*ETEC_2065*/*ETEC_2066*	*yedR*/ETEC-specific
2458348	**TGTGA**TCTGAA**TC**T**CA**	*ETEC_2278*	***cdd***
	**TG**C**GA**TGCGTCG**C**G**CA**		
2492757	AT**TGA**TCGCCC**TCACA**	*ETEC_2309*	*yeiQ*
2555083	CG**TGA**CCAAAG**TCTCA**	*(ETEC_2360)*	*(yfaQ)*
2729713	TT**TGA**AGCTTG**TCACA**	*ETEC_2510*/*ETEC_2511*	*mntH*/*nupC*
2735124	A**GT**T**A**TTCATG**TCAC**G	*ETEC_2514*	*yfeC*
2795423	**TGTGA**GCCATGA**CACA**	*(ETEC_2572)/ETEC_2573*	***(aegA)/narQ***
2810983	C**GTGA**TCAAGA**TCACA**	*ETEC_2586*	***hyfA***
2887131	**T**T**TGA**TCTCGC**TCACA**	(*ETEC_2666*)/*ETEC_2665*	(***xseA***)/***guaB***
3012645	**TGTGA**TCCCCACA**ACA**	(*ETEC_2793*)	(***ung***)
3048307	TT**TGA**CGAGCA**TCACC**	*(ETEC_2822)*	*(emrB)*
3132920	G**GTGA**CCGGTT**TCACA**	*ETEC_2905*/*ETEC_2906*	*ascG* /*ascF*
3161660	**TGTGA**CCGTGG**TC**G**CA**	(*ETEC_2933*)	(***nlpD***)
3184337	CG**TGA**TGCGTG**T**A**ACA**	*(ETEC_2956)/ETEC_2955*	*(cysI)/cysH*
3196088	**TGTGA**TTACGA**TCACA**	*ETEC_2966*/*ETEC_2967*	*ygcW*/*yqcE*
3223792	A**GTGA**TCTTGA**TC**T**CA**	*ETEC_2986*	*sdaC*
	A**GT**T**A**TGTATC**T**AT**CA**		
3234980	**TG**C**GA**TCGTTA**TCACA**	(*ETEC_2994*)/*ETEC_2995*	(*fucU*)/*fucR*
3265047	**TGTGA**CCTGGG**TCAC**G	*ETEC_3017*	*rppH*
3324543	**TGTG**GGCTACG**T**A**ACA**	*(ETEC_3075)*	*(ydhD)*
3361162	n.d.	*ETEC_3105*	***serA***
3368992	**T**T**TGA**TGCACCG**CACA**	(*ETEC_3113*)	(*ygfI*)
3382158	**TGTGA**TCTACAA**CAC**G	*ETEC_3126*	*cmtB*
3390811	**TGTGA**TTTGCT**TCACA**	*ETEC_3133*	***galP***
3408173	**TGTGA**TGTGGA**T**A**ACA**	*ETEC_3154*	***nupG***
3442697	**TGTGA**TGATTG**TC**G**CA**	*ETEC_3186*	ETEC-specific
3558573	A**GTGA**TTTGGC**TCACA**	*ETEC_3291*	*ygiS*
3580767	A**GTGA**CTTGCA**TCACA**	(*ETEC_3318*)	(*yqiH*)
3635301	AT**TGA**TCTAAC**TCAC**G	*ETEC_3362*	***uxaC***
3642302	CT**TGA**AGTGGG**TCACA**	*(ETEC_3372)*	*(yqjG)*
3665634	**TGTGA**TCAATG**TCA**AT	*ETEC_3393*/*ETEC_3394*	*garP*/*garD*
	**TGTG**CTTTAGCG**C**G**CA**		
3721308	G**GTGA**TTGATG**TCAC**C	(*ETEC_3446*)	(*greA*)
3785700	CG**TG**GGTCGCA**TCACA**	*(ETEC_3510)*	*(mreC)*
3878729	G**GTGA**TTTTGA**TCAC**G	*ETEC_3614*/*ETEC_3615*	*ppiA* /*tsgA*
3908574	G**GTGA**TCGCGC**TCACA**	(*ETEC_3645*)	(***hofM***)
3918861	**TGTGA**GTGGAA**TC**G**CA**	*ETEC_3652/ETEC_3653*	*yhgE/pck*
3986400	C**GTGA**TTTTATC**CACA**	*ETEC_3707*	***rpoH***
4105040	AG**T**A**A**GGCAAG**TC**C**C**T	n.a.	*n.a.*
4111116	**TGTGA**CGGGGC**T**A**ACA**	(*ETEC_3806*)	(*wecH*)
4153055	**TGTGA**TCTGAA**TCACA**	*ETEC_3840*	***yibI***
	**TGTGA**TCTACAG**CA**TG		
4153191	**TGTGA**TTGATA**TCACA**	*ETEC_3841*	*mtlA*
	**TGTGA**TGAACG**TCAC**G		
4158433	n.d.	*ETEC_3846*	*lldP*
4196869	**TG**CA**A**TCGATA**TCACA**	*ETEC_3886*	*dinD*
4251326	CT**T**ACTCCTGC**TCACA**	*ETEC_3938*	ETEC specific
4266125	G**GTGA**TGGCATC**C**G**C**G	(*ETEC_3956*)	(*nepI*)
4290730	GG**TGA**GCAAAAC**CAC**G	*(ETEC_3979)*	*(yidR)*
4322430	AT**TGA**CCTGAG**TCACA**	*(ETEC_4010)*	*(yieL)*
4340544	CT**TGA**CCACGG**TCA**G**A**	*(ETEC_4025)/ETEC_4024*	*(atpA)/atpG*
4344649	**TGTGA**TCTGAAG**CAC**G	*ETEC_4030*	*atpI*
4373517	**TGT**A**A**TGCTGG**T**A**ACA**	*(ETEC_4051)*	*(ilvG)*
4402013	C**GTG**CTGCATA**TCAC**G	*(ETEC_4077)*	*(rffM)*
4412999	C**GTGA**TCAATT**T**A**ACA**	*ETEC_4085*/*ETEC_4085*	*hemC* /*cyaA*
4438352	G**GTGA**TGAGTA**TCAC**G	*ETEC_4107*/*ETEC_4108*	*ysgA* /*udp*
	**TGTGA**TTTGAA**TCAC**T		
4508745	**TGTGA**TATTTG**TCACA**	(*ETEC_4165*)/*ETEC_4164*	(*fdhD*)/*fdoG*
4517442	C**GTGA**TCGCTG**TC**C**CA**	(*ETEC_4173*)	(*rhaA*)
4564670	**TG**C**GA**TCCGCC**TCA**T**A**	*ETEC_4216*/*ETEC_4217*	*ptsA*/*frwC*
4668870	**TGT**A**A**CAGAGA**TCACA**	*ETEC_4289/ETEC_4290*	***malE/malK***
4725047	**TGTG**CGGATGA**TCACA**	n.a.	n.a.
4731402	**TGTGA**TCTTGCG**CA**T**A**	(*ETEC_4365*)	(*aphA*)
4761367	C**GTGA**TGGCTG**TCAC**G	*ETEC_4389*	*fdhF*
4846352	n.d.	*ETEC_4464*	ETEC-specific
4848117	C**GTGA**GTTCTG**TCACA**	n.a.	n.a.
4863253	**T**T**TGA**TCAACA**TC**G**CA**	(*ETEC_4478*)	(ETEC-specific)
4873926	G**GTGA**TCTATT**TCACA**	*ETEC_4486*/*ETEC_4487*	*aspA*/*fxsA*
4930149	**TGTGA**TGAACT**TCA**A**A**	*ETEC_4545*/*ETEC_4546*	*yjfY*/*rpsF*
4940903	**TGTGA**TCACTA**TC**G**CA**	*ETEC_4557*/*ETEC_4558*	ETEC-specific/*ytfA*
4993073	**TGTGA**CTGGTA**TC**T**C**G	(*ETEC_4604*)	(*valS*)
5002854	**TGT**A**A**CCTTTG**TCACA**	*ETEC_4610/tRNA-Leu*	*yjgB/tRNA-Leu*
5030724	**TG**C**GA**TGAATG**TCACA**	*ETEC_4633*/*ETEC_4634*	*gntP* /*uxuA*
5129400	CG**T**ACCGTCGG**TCACA**	*(ETEC_4736)*	*(yjjI)*
5129944	**TGTGA**TGTATA**TC**GA**A**	*ETEC_4736*/*ETEC_4737*	*yjjI*/*deoC*

a Chomosome coordinate of the ChIP-seq peak in H10407. Underlined text indicates that the ChIP-seq peak maps to sequence that is not conserved in *E. coli* K-12.

b CRP binding site sequence predicted by MEME. “n.d.” indicates that MEME did not detect a putative binding site.

c Genes in parentheses indicate that the ChIP-seq peak is located within that gene. Downstream genes are only listed if the annotated gene start is ≤300 bp downstream of the CRP ChIP-seq peak. “n.a.” indicates that no genes starts are ≤300 bp from the CRP ChIP-seq peak.

d *E. coli* K-12 homologues are listed for the ETEC genes in the previous column. Genes in parentheses indicate that the ChIP-seq peak is located within that gene. “n.a.” indicates that no genes starts are ≤300 bp from the CRP ChIP-seq peak. “ETEC-specific” indicates that there is no K-12 homologue. Underlined genes have been identified as CRP targets in a previous ChIP-chip study [15]. Bold genes are listed as CRP targets in the Ecocyc database.

### Unoccupied high-affinity CRP binding targets on p948 and p666 are bound by H-NS

**Figure 2:**
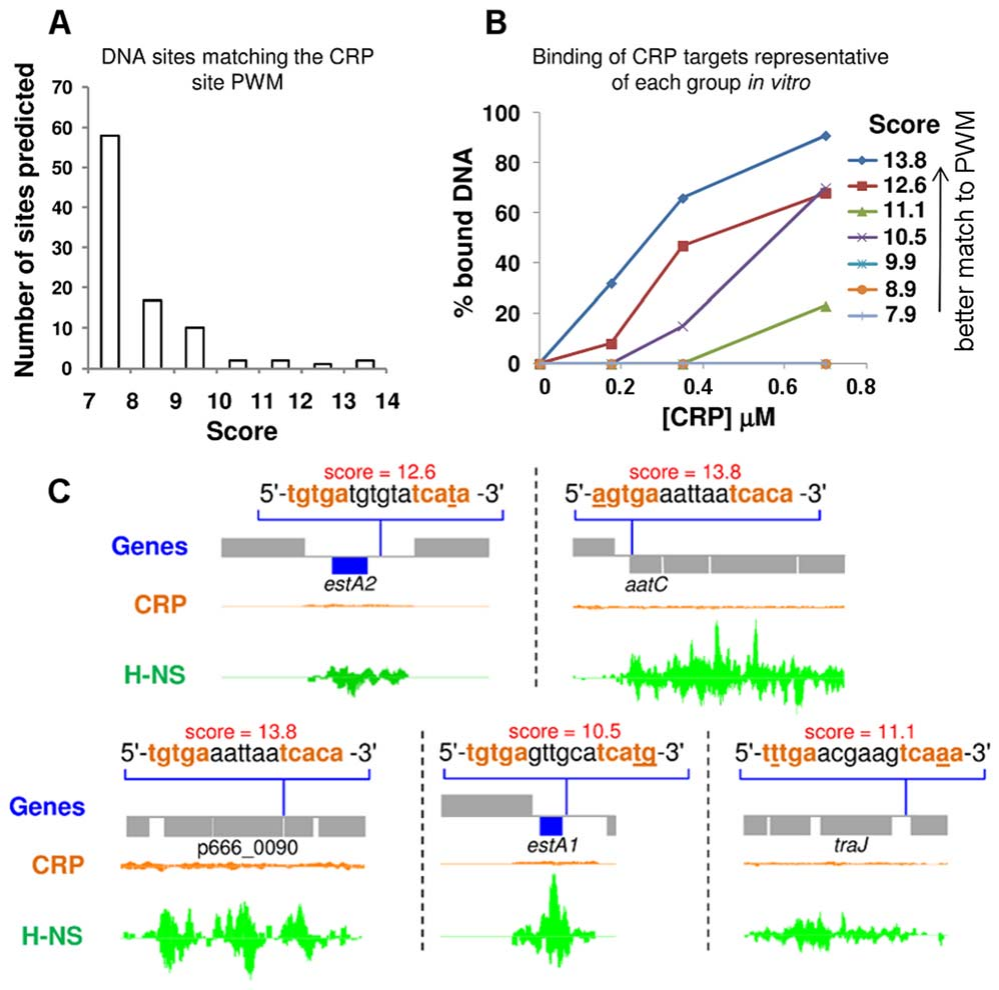
Unoccupied CRP sites on p666 and p948 align with H-NS bound regions. A) A histogram showing the number of putative CRP binding sites in each of 7 discrete bins. Each bin is delineated by the “score” of the putative CRP site. A high score indicates a better match to the Position Weight Matrix that represents the consensus for CRP binding. B) The graph illustrates binding of CRP to a target from each of the bins shown in Panel A. CRP was used at concentrations of 0, 175, 350 or 700 nM. C) ChIP-seq data for CRP and H-NS binding at five regions of plasmids p666 and p948 that contain unoccupied CRP targets bound by CRP *in vitro*. The CRP and H-NS binding profiles are plots of sequence read counts at each position of the genome on both the top (above the central line) and bottom (below the central line) strand of the DNA. The y-axis scale is the same in each panel. The scale for H-NS binding is 1,785 reads on each strand and for CRP binding is 14,000 reads on each strand.

To better understand the lack of CRP binding to p948 and p666 we took a bioinformatic approach. CRP targets were aligned to generate a position weight matrix (PWM). The PWM was then used to search p948 and p666 for CRP sites. A continuum of over 100 potential CRP targets was identified. However, we recognise that the vast majority of these are likely to be false positives. Hence, we next sought to differentiate between genuine CRP sites and spurious predictions. To do this, predicted sites were scored, grouped, and ranked on the basis of their match to the PWM (Fig. 2A, S1 Table). Electrophoretic mobility shift assays (EMSA) were then used to measure binding of CRP to a target from each group so that a meaningful cut-off could be established. The result is illustrated graphically in Fig. 2B. The raw data are shown in S1A Fig. We found that predicted sites with a score<10 did not bind CRP. To assess the affinity of CRP for all predicted targets scoring >10 a second set of EMSA experiments was done (S1B Fig.). Hence, we identified a total of 5 potential CRP targets on p666 and p948. Interestingly, the *estA1* and *estA2* genes, which both encode ST, were amongst the 5 targets (Fig. 2C). Remarkably, all 5 of the plasmid borne CRP targets identified *in silico*, and bound tightly by CRP *in vitro*, were occupied by H-NS *in vivo* (Fig. 2C).

### The *estA2* gene is transcribed from a Class I CRP dependent promoter

**Figure 3:**
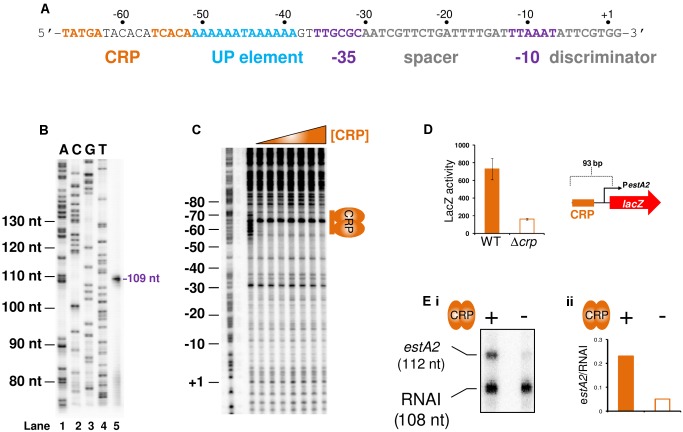
The *estA2* promoter is activated by a Class I CRP dependent mechanism. A) Sequence of the *estA2* gene regulatory region. The CRP binding site is shown in orange, the UP element is blue and the promoter -10 and -35 elements are shown in purple. The different promoter positions are numbered relative to the transcription start site (+1). B) Location of the P*estA2* transcription start site. The gel shows the product of an mRNA primer extension analysis to determine the *estA2* transcription start site (Lane 5). The gel was calibrated using arbitrary size standards (A, C, G and T in Lanes 1–4). C) Binding of CRP to P*estA2*. The panel shows the result of a DNAse I footprint to monitor binding of CRP to the 93 bp P*estA2* DNA fragment. The gel is calibrated with a Maxim-Gilbert DNA sequencing reaction. CRP was added at concentrations of 0.35–2.1 µM. D) CRP is required for transcription from P*estA2 in vivo*. The panel shows a cartoon representation of the 93 bp P*estA2::lacZ* fusion and a bar chart illustrates LacZ activity in lysates of cells carrying this fusion. Assays were done in LB medium. E) i) Stimulation of P*estA2* by CRP *in vitro*. The figure shows the results of an *in vitro* transcription reaction. The 112 nt transcript initiates from P*estA2* and the 108 nt RNAI transcript is an internal control. CRP was added at a concentration of 350 nM and RNA polymerase was added at a concentration of 400 nM. ii) quantification of band intensities from the *in vitro* transcription analysis.

To understand if CRP could regulate ST production we focused first on *estA2*. This derivative of the toxin is more commonly associated with human disease and ETEC H10407 is somewhat unusual in also encoding *estA1* [24]. The sequence of the *estA2* regulatory region is shown in Fig. 3A. A 93 bp DNA fragment, containing the regulatory region, was cloned into the *lacZ* reporter plasmid pRW50 to generate a *lacZ* fusion (S2A Fig.). The *estA2* TSS was then determined using mRNA primer extension analysis. We detected a single extension product, of 109 nucleotides (nt) in length (Fig. 3B). The position of the TSS is labelled “+1” in Fig. 3A. Promoter -10 (5′-TTAAAT-3′) and -35 (5′-TTGCGC-3′) elements were observed at the expected positions upstream of the TSS. Throughout this work we refer to this promoter, highlighted purple in Fig. 3A, as P*estA2*. To confirm CRP binding at the predicted site we used DNase I footprinting (Fig. 3C). As expected, CRP protected the predicted target from digestion. Additionally, CRP induced DNase I hypersensitivity in the centre of the site. Note that the CRP site is centred 59.5 bp upstream of the TSS and adjacent to an AT-rich sequence that may be an UP element (Fig. 3A). Thus, we hypothesised that P*estA2* is a class I CRP activated promoter. To test our hypothesis we first determined whether CRP could indeed activate P*estA2*. To do this, we compared LacZ expression in M182Δ*lac* and M182Δ*lac*Δ*crp* cells carrying the P*estA2::lacZ* fusion. The data show that loss of CRP results in a 3-fold decrease in LacZ expression from P*estA2* (Fig. 3D). We next tested the ability of CRP to activate P*estA2 in vitro*. The 93 bp DNA fragment was cloned upstream of the λ*oop* terminator in plasmid pSR. In the context of this construct a 112 nt transcript is generated by RNA polymerase from P*estA2 in vitro*. The amount of transcript can then be quantified by electrophoresis. The result of the analysis, with and without CRP, is shown in Fig. 3E. As expected, an intense band corresponding to the 112 nt transcript was observed. Production of the transcript was stimulated by CRP. Note that CRP had no effect on production of the 108 nt control RNAI transcript from the plasmid replication origin. Finally, we examined the AT-rich DNA sequence (highlighted blue in Fig. 3A) located between the CRP site and the promoter -35 element. We found that increasing the GC content of the putative UP-element altered migration of the 93 bp DNA fragment on an agarose gel, consistent with a change in DNA topology (S3A Fig.). Moreover, these changes to the UP-element rendered P*estA2* insensitive to CRP *in vivo* and *in vitro* (S3B Fig.).

### H-NS excludes CRP from the *estA2* promoter and represses *estA2* transcription

**Figure 4:**
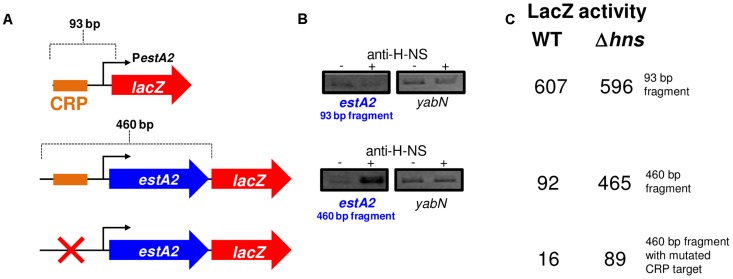
The *estA2* promoter is repressed by H-NS. A) The panel shows different P*estA2::lacZ* fusions. The *lacZ* gene is shown as a red arrow and the *estA2* gene is shown as a blue arrow. P*estA2* is illustrated using a bent arrow and the CRP binding site is shown as an orange box. B) H-NS binds to P*estA2* only in the presence of flanking DNA. ChIP-PCR was used to measure binding of H-NS to the different P*estA2* derivatives cloned in pRW50. PCR products were generated using primers that could detect P*estA2* in the context of both the 93 bp fragment and the longer 460 bp fragment. C) The values are β-galactsidase activity values for lysates of M182, or M182Δ*hns*, carrying the different P*estA2* derivatives. Assays were done in LB medium.

Promoters can be liberated from H-NS repression if separated from flanking, H-NS bound, DNA [25]. We reasoned that this might be why, when isolated on the 93 bp fragment, P*estA2* was active and dependent on CRP. To test this logic we generated a further two P*estA2::lacZ* fusions using the pRW50 plasmid system. The additional P*estA2* DNA fragments were both 460 bp in length and include the full *estA2* gene that was entirely bound by H-NS in our ChIP-seq assay (Fig. 2C). The CRP site was ablated in one of the additional fragments by introducing point mutations that are predicted to disrupt CRP binding. The sequence of the DNA fragments is shown in S2A Fig. The *lacZ* fusions are illustrated graphically in Fig. 4A. Our expectation was that the longer 460 bp fragment would bind H-NS whilst the starting 93 bp fragment would not. To test this prediction we used ChIP. Thus, we compared H-NS binding to the different P*estA2* containing fragments *in vivo*. Fig. 4B shows results of a PCR analysis to measure enrichment of the P*estA2* locus. As expected, P*estA2* was only enriched in anti-H-NS immunoprecipitates when in the context of the 460 bp fragment. Crucially, enrichment is specific because, in a set of control PCR reactions, there was no enrichment of the *yabN* locus in any immunoprecipitate.

Our ChIP analysis suggests that the 460 bp fragment containing P*estA2* is subject to regulation by H-NS. To confirm that this was the case, the various pRW50 derivatives were used to transform M182Δ*lac* and M182Δ*lac*Δ*hns* cells. We then measured LacZ activity, driven by P*estA2*, in the transformants. Consistent with our expectations the data show that P*estA2* is repressed 5-fold by H-NS only in the context of the 460 bp DNA fragment (Fig. 4C). Importantly, mutations in the CRP binding site abolish P*estA2* activity in the absence of H-NS. Hence, the measured LacZ expression must be driven by P*estA2* rather than any spurious promoters located within the *estA2* gene. Taken together our ChIP-seq and LacZ activity data show that H-NS prevents CRP from activating P*estA2*.

### The *estA2* and *estA1* promoters are differently regulated by CRP but similarly regulated by H-NS

**Figure 5:**
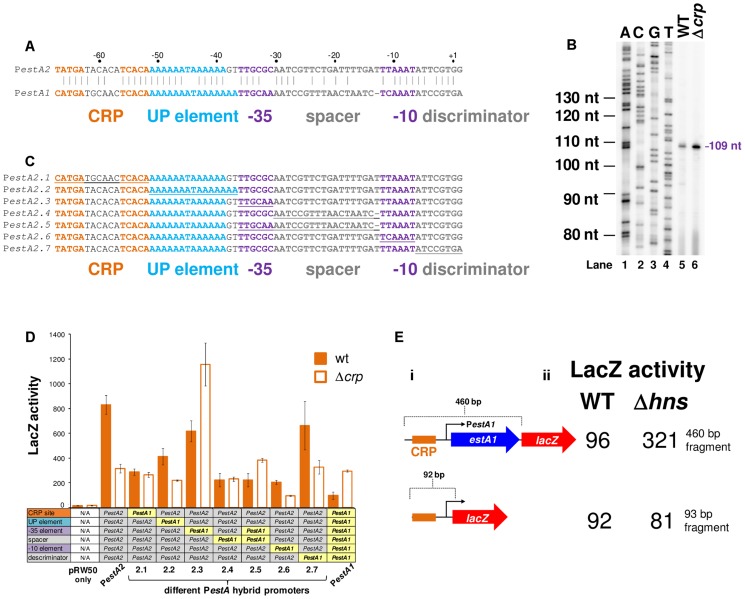
Comparison of P*estA1* and P*estA2* reveals differential activity and regulation by CRP. A) Comparison of P*estA1* and P*estA2*. The panel shows the DNA sequences of P*estA1* and P*estA2*. Bases that are identical are highlighted by a solid vertical line. The CRP sites are shown in orange, the UP element in blue and the core promoter elements in purple. The sequences are numbered with respect to the transcription start site (+1). B) Location of the P*estA1* transcription start site. The gel shows products from an mRNA primer extension analysis (Lanes 5 and 6). The gel was calibrated using arbitrary size standards (A, C, G and T in Lanes 1–4). C) Sequences of hybrid *estA* promoters. The sequences labelled *estA2.1* through *estA2.7* are derivatives of the 93 bp P*estA2* DNA fragment where different sequence elements have been replaced with the equivalent sequence from P*estA1*. D) The bar chart shows β-galactosidase activity measurements for lysates obtained from cultures of M182, or the Δ*crp* derivative, containing the indicated hybrid promoter fragment was fused to *lacZ*. E) The panel shows different P*estA1::lacZ* fusions. The *lacZ* gene is shown as a red arrow and the *estA1* gene is shown as a blue arrow. P*estA1* is illustrated using a bent arrow and the CRP binding site is shown as an orange box. Assays were done in LB medium.

The *estA1* regulatory region, located on plasmid p666, contains a sequence similar to P*estA2* (Fig. 5A). We expected that this sequence would be the *estA1* promoter (P*estA1*). To test this expectation we created a 92 bp P*estA1::lacZ* fusion, equivalent to the 93 bp P*estA2::lacZ* fusion described above, and mapped the 5′ end of the resulting mRNA. As expected, the primer extension product was 109 nt in length (Fig. 5B). Hence, Pe*stA1* and P*estA2* use equivalent TSSs. However, we were surprised that the intensity of the P*estA1* primer extension product *increased* in cells lacking CRP (Fig. 5B). Closer examination of the alignment in Fig. 5A shows that, whilst P*estA1* and P*estA2* are similar, there are differences in the sequence and position of key promoter elements. To try and understand which changes result in the aberrant behaviour of P*estA1* we made a set of hybrid promoters. The hybrid constructs are derived from the CRP-activated *estA2* promoter. In each hybrid, named P*estA2.1* through P*estA2.7*, a region of P*estA2* was replaced with the equivalent region from P*estA1* (see underlined sequences in Fig. 5C). The ability of the different hybrid promoters to drive *lacZ* expression, with and without CRP, was then tested. The results are shown in Fig. 5D. Note that, in Fig. 5D, the composition of each hybrid promoter is indicated in the grid below the graph. For example, P*estA2.1* is derived from P*estA2* but contains the P*estA1* CRP site. As expected, both P*estA1* and P*estA2* were able to drive *lacZ* expression but CRP had opposite effects. Moreover, maximal expression from P*estA1* was 3-fold lower than from P*estA2*. Only P*estA2.3* and P*estA2.5*, which both carried the same changes in the promoter -35 element, exhibited a reversed dependence on CRP. Hence, the P*estA1* -35 element must be responsible for the altered CRP dependence. All other hybrid promoters exhibited an overall reduction in activity compared to the parent P*estA2* construct. We conclude that this combination of changes results in the lower activity of P*estA1*. Note that both P*estA1* and P*estA2* were bound by H-NS in our ChIP-seq analysis (Fig. 2C). We reasoned that cloning P*estA1*, with flanking DNA, would reveal H-NS mediated repression. We generated a derivative of the P*estA1::lacZ* fusion where the downstream boundary was extended to include the entire *estA1* gene (S2B Fig., Fig. 5Ei). As expected, transcription from P*estA1* was repressed by H-NS in the presence of downstream DNA (Fig. 5Eii).

### The *eltAB* operon is indirectly repressed by CRP and directly repressed by H-NS

**Figure 6:**
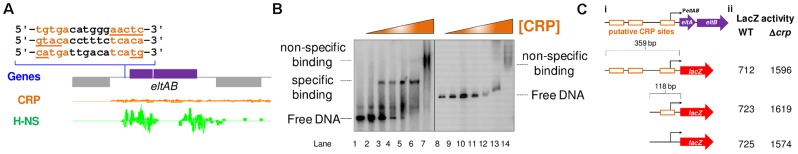
The *eltAB* promoter is indirectly repressed by CRP. A) The Panel shows ChIP-seq data for CRP and H-NS binding at the *eltAB* locus. The sequence of 3 putative CRP binding sites proposed by Bodero and Munson (2009) are shown. The CRP and H-NS binding profiles are plots of sequence read counts at each position of the genome on both the top (above the central line) and bottom (below the central line) strand of the DNA. The y-axis scale for H-NS binding is 1,785 reads on each strand and for CRP binding is 14,000 reads on each strand. B) Results of an Electorphoretic Mobility Shift Assay to measure binding of CRP to the 93 bp P*estA2* fragment (Lanes 1–7) or the 359 bp P*eltAB* fragment (Lanes 8–14). Specific and non-specific binding of CRP is indicated to the left and right of the gel. CRP was added at a concentration of 0.2–7.0 µM. C) Panel (i) shows different P*eltAB::lacZ* fusions. The *lacZ* gene is shown as a red arrow and the *eltAB* operon is shown in purple. P*eltAB* is illustrated using a bent arrow and the putative CRP binding sites are shown as open orange boxes. In panel (ii) the values are β-galactsidase activity measurements taken in M182 or the Δ*crp* derivative. Assays were done in LB medium.

We next turned our attention to the LT toxin promoter (P*eltAB*) [26], [27]. Previously, Bodero and Munson [27] showed that transcription from this promoter was repressed by CRP. A mechanism for repression was proposed whereby CRP acted directly by binding three DNA targets overlapping P*eltAB* [27]. Even so, no CRP binding at P*eltAB* was identified by our ChIP-seq analysis (Fig. 6A). It is possible that this is because H-NS also excludes CRP from this locus (Fig. 6A). However, we also failed to identify CRP targets at P*eltAB* in our bioinformatic screen, even below the stringent cut-off (Fig. 2, S1 Table). In retrospect, this appears to be because all of three P*eltAB* CRP binding sites contain at least 4 mismatches to the consensus for CRP binding (Fig. 6A). Hence, we measured the affinity of CRP for P*eltAB* using EMSA assays. In parallel, we tested CRP binding to P*estA2* as a control. As expected, CRP bound tightly to P*estA2* at low concentrations (Fig. 6B, lanes 1–6). At high CRP concentrations further non-specific binding was observed (evidenced by a conspicuous “smear” in DNA migration in lane 7). In the equivalent experiment, with P*eltAB*, no specific binding of CRP was observed (lanes 8–13). However, non-specific CRP binding was again detectable at high protein concentrations (lane 14). Hence, CRP does not bind specifically to P*eltAB*. We hypothesised that previously observed changes in P*eltAB* activity, in cells lacking CRP, may occur indirectly. To test this, we cloned a 359 bp DNA fragment, containing P*eltAB*, into our pRW50 *lacZ* expression system. We also made a truncated 118 bp derivative of this construct where two of the three putative CRP targets were removed. A derivative of the truncated 118 bp construct, where the remaining CRP site was completely ablated by point mutations, was also made. The DNA sequences of the different constructs are shown in S2C Fig. They are illustrated graphically in Fig. 6Ci. Consistent with previous measurements, we found that transcription from P*eltAB* increased 2.5 fold in the absence of CRP. However, the response of P*eltAB* was identical when the CRP binding sites were removed (Fig. 6Cii). Hence, although CRP represses transcription from P*eltAB*, this must occur indirectly.

**Figure 7:**
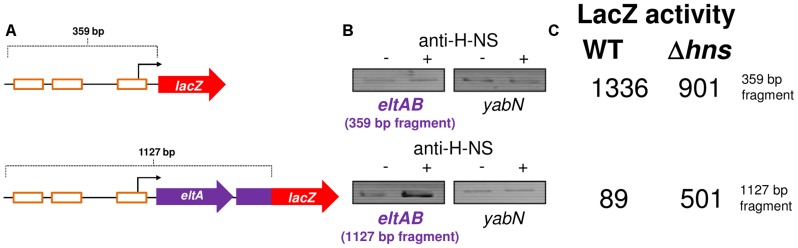
The *eltAB* promoter is directly repressed by H-NS. A) The panel shows two P*eltAB::lacZ* fusions. B) The results of a ChIP-PCR analysis used to measure binding of H-NS to the two different P*eltAB* derivatives shown in Panel A. C) The values are β-galactsidase activity measurements for lysates of M182, or the Δ*hns* derivative, carrying the different P*estA2::lacZ* fusions. Assays were done in LB medium.

Given the configuration of H-NS binding at the *eltAB* locus (Fig. 6A) we reasoned that P*eltAB* would be repressed by H-NS in the presence of sufficient flanking DNA. As we had done previously for P*estA1* and P*estA2*, we compared the binding of H-NS to P*eltAB* in the presence and absence of the downstream flanking sequence. The different DNA constructs are illustrated in Fig. 7A and results of ChIP experiments to measure H-NS binding are shown in Fig. 7B. As predicted, enrichment of P*eltAB*, in immunoprecipitations with anti-H-NS, was only observed in the presence of downstream DNA. Importantly, this enrichment was specific to P*eltAB* and not observed for the control locus *yabN*. Corresponding LacZ activities, for the different DNA constructs, measured in M182 or the Δ*hns* derivative, are shown in Fig. 7C. Incorporation of flanking DNA downstream of P*eltAB* resulted in a 15-fold reduction in LacZ activity that was largely relieved in the absence of H-NS.

### CRP and H-NS allow the *estA1*, *estA2* and *eltAB* promoters to respond to glucose and salt

**Figure 8:**
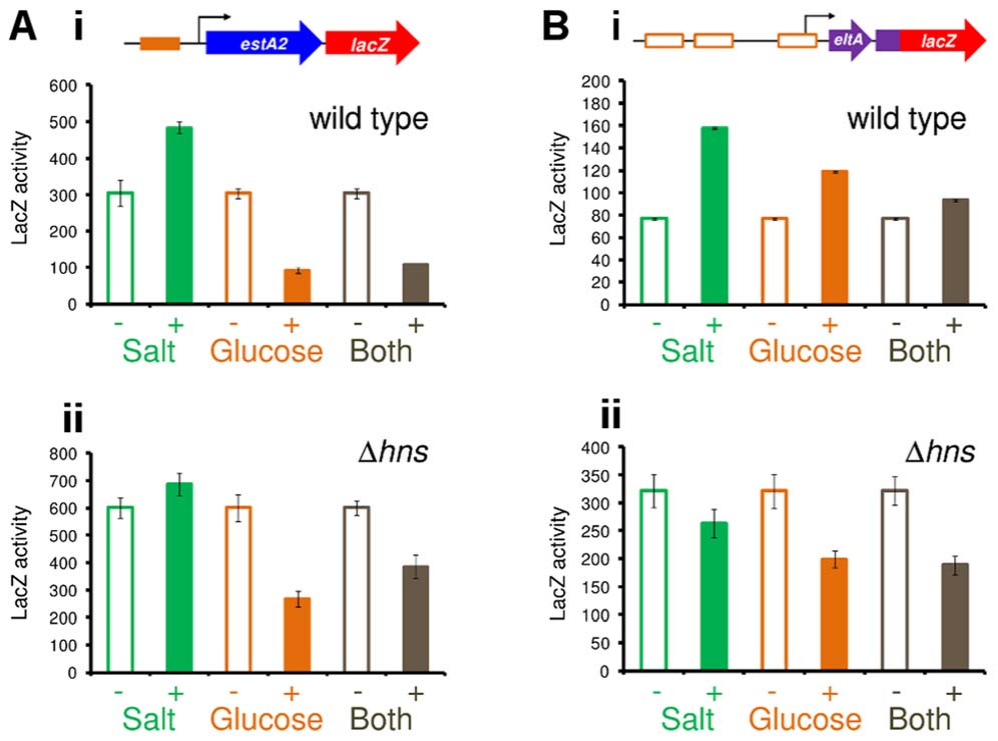
H-NS and CRP integrate signals of osmolarity and metabolism to control expression of LT and ST. The figure shows β-galactosidase activity measurements for lysates obtained from cultures of M182 (i) or the Δ*hns* derivative (ii) containing A) P*estA2* or B) P*eltAB* fused to *lacZ* in plasmid pRW50. Cultures were grown in the presence and absence of 2% glucose and/or salt (60 mM NaCl and 20 mM KCl). Assays were done in M9 minimal medium so that the glucose and salt concentrations could be more accurately controlled.

Given the established regulatory connections between CRP and glucose, and between H-NS and salt, we next measured changes in the activity of P*estA1*, P*estA2* and P*eltAB* in response to glucose and salt. A complete description of assay conditions is provided in the Materials and Methods section. Briefly, to establish the range of conditions across which the promoters were able to respond, we examined the effect of titrating glucose or salt into the growth medium individually. In all experiments, we used the promoter::*lacZ* fusions that included downstream flanking DNA. This was to ensure that signals sensed by both CRP and H-NS could be integrated. As expected, the activity of P*estA1* was low. Consequently, the effects of glucose and salt were negligible (S4A Fig.). Conversely, the activity of P*estA2* was sensitive to both glucose and salt (S4B Fig.). Thus, *lacZ* expression driven by P*estA2* was repressed by glucose (orange line) and enhanced by salt (green line). As expected, P*eltAB* activity increased in the presence of both salt and glucose, but induction by salt was more prominent (S4C Fig.). We hypothesised that, for P*estA2*, the inhibitory effect of glucose should override the stimulatory effect of salt. Our reasoning was that, although H-NS can repress P*estA2*, the promoter is ultimately dependent on CRP for activity. Hence, we examined the effect of adding salt and glucose, to cells carrying the P*estA2::lacZ* fusion, separately and in combination (Fig. 8A). As predicted, the inhibitory effect of glucose was dominant (Fig. 8Ai) and was still observed in the absence of H-NS (Fig. 8Aii). Conversely, the stimulatory effect of salt required H-NS (compare green bars in Fig. 8). Importantly, in a separate experiment, we also showed that the effect of glucose on P*estA2* activity requires that the CRP site is intact (S4D Fig.). The combined effect of salt and glucose on P*eltAB* was more difficult to predict because CRP acts via an undefined, and indirect, mechanism. The result of the analysis (Fig. 8B) shows that the stimulatory effects of salt and glucose on transcription from P*eltAB* are not additive. Moreover, the stimulatory effect of glucose requires H-NS.

### The response of P*eltAB* and P*estA2* to CRP and H-NS is conserved in other ETEC isolates and during host cell attachment

**Figure 9:**
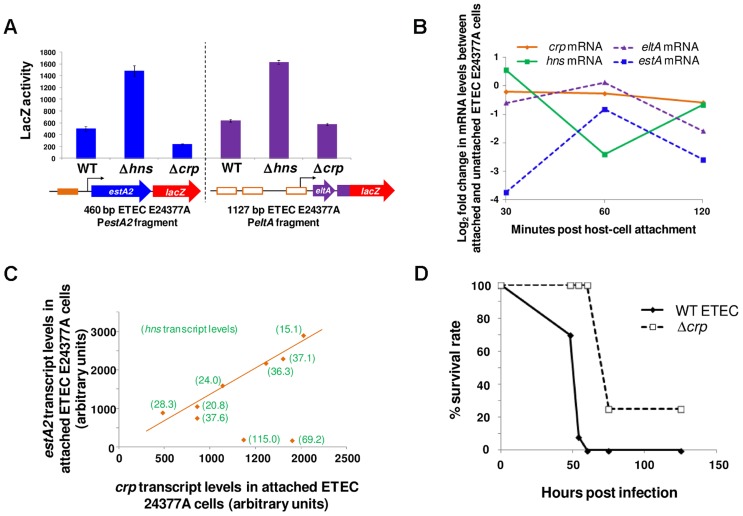
Modulation of *estA2* and *eltA* transcription during attachment of ETEC E24377A to gut epithelial cells. A) The figure shows β-galactosidase activity measurements for lysates obtained from cultures of M182 or the Δ*hns* and Δ*crp* derivatives containing P*estA2* (460 bp fragment) or P*eltAB* (1126 bp fragment) from ETEC 24377A fused to *lacZ* in plasmid pRW50. B) The panel shows log_2_ fold changes in the transcription of *crp*, *hns*, *eltA* and *estA* in ETEC E24377A cells over a two hour incubation with a Caco-2 intestinal epithelial cell culture (29). The log_2_ values represent the fold change in transcription between ETEC cells attached and unattached to Caco-2 cells at each time point. C) The panel shows a scatter plot of absolute *crp* and *estA2* mRNA levels in ETEC E24377A attached to Caco-2 intestinal epithelial cells. Each data point represents a different biological replicate. For each data point the absolute level of *hns* mRNA is shown in parenthesis. D) The panel shows the survival rate of BALB/C mice (n = 30) after intranasal inoculation with wild type ETEC H10407 or the Δ*crp* derivative.

Examination of all sequenced ETEC genomes reveals slight variations in the sequence of the *eltAB* and *estA2* promoter sequences (recall that ETEC H10407 is somewhat anomalous in also encoding *estA1*). Thus, we next sought to understand if our model for regulation of LT and ST expression was broadly applicable. We focused our efforts on ETEC E24377A since i) the genome has been sequenced and ii) a vast array of independently generated transcriptomic data are available for this organism [28], [29]. Using ETEC E24377A DNA as a template, we generated a 460 bp P*estA2*, and 1126 bp P*eltAB* DNA fragment. The sequences are shown in S2D Fig. The DNA fragments were cloned into pRW50 and the ability of the promoters to drive *lacZ* expression in response to CRP and H-NS was measured. As expected, transcription from P*estA2* was repressed by H-NS and activated by CRP whilst P*eltAB* was repressed by H-NS (Fig. 9A). We observed no effect of CRP on transcription from *PeltAB* in the context of the 1126 bp ETEC E24377A fragment. This is not unexpected because CRP acts indirectly and these indirect CRP effects have only previously been observed in the context of short DNA fragments containing P*eltAB* that are not subject to direct repression by H-NS. We note that Sahl and Rasko previously examined the global transcriptome response of E24377A to glucose levels and bile salts [28]. In exact agreement with our model for toxin regulation, and the data in Fig. 9A, this study confirmed that i) salt induced expression of both toxins and ii) glucose inhibited expression of *estA2* [28]. Fortuitously, changes in the ETEC E24377A transcriptome, prompted by ETEC attachment to human gut epithelial cells, have also been quantified comprehensively [29]. Briefly, in these experiments, ETEC were added to sets of Caco-2 intestinal epithelial cell tissue cultures. Over a time course, ETEC that had adhered to host cells were separated from non-adhered ETEC. The transcriptomes of adhered and non-adhered ETEC were then compared. By mining these data, we next sought to determine if our model was consistent with observed changes in the transcription of *crp*, *hns*, *eltA* and *estA* during host cell attachment. Briefly, our data predict that changes in *estA* expression should be directly correlated to changes in the level of CRP and inversely correlated with changes in levels of H-NS. Conversely, levels of *eltA* expression should be inversely correlated with levels of H-NS. The result of the analysis is illustrated in Fig. 9B. The data show that the relative levels of *crp* transcription in attached and unattached cells are similar (orange line). However, levels of *hns* transcription change dramatically (green line) 60 minutes after host cell attachment. As predicted by our model, levels of *estA2* and *eltA* transcription (dashed lines) inversely track changes *hns* transcript levels. When undertaking this analysis we noticed that, although there was little change in the relative level of *crp* mRNA between attached and unattached ETEC cells, the absolute level of *crp* mRNA did fluctuate across the time course of the experiment and between biological replicates. Strikingly, when these absolute mRNA levels are compared there is a clear linear relationship between *crp* and *estA2* expression (Fig. 9C). Note that in Fig. 9C the absolute level of *hns* mRNA has been added in parenthesis for each data point. Remarkably, the only two outlying data points in this plot correspond to the two samples with increased *hns* expression. We conclude that regulation of *estA2* and *eltA* by CRP and H-NS is important during the attachment of ETEC to human intestinal epithelial cells, and that the regulatory control of ETEC toxins is conserved across different strains.

### Disrupting the regulatory switch attenuates ETEC virulence

Taken together, our data suggest that CRP and H-NS form a regulatory switch that controls ETEC toxicity. We next sought to examine the effect of disabling the switch on virulence. This is not straightforward because no animal model faithfully mimics the disease caused by ETEC in humans. However, intranasal mouse models have been used as a proxy for measuring *E. coli* pathogenicity [30]. Importantly, pathogenic *E. coli* cause more severe disease in this model than non-pathogenic strains [30]. Furthermore, ETEC strains lacking genes encoding toxins and known colonisation factors are less virulent in this model [31]. We opted to disrupt the regulatory switch by removing the *crp* rather than the *hns* gene. This was a deliberate decision since *E. coli* strains lacking *hns* are severely attenuated for growth in laboratory conditions. Conversely, the *crp* null derivative of ETEC H10407 was only mildly compromised for growth in liquid culture. Hence, we compared pathogenicity of ETEC H10407, and the *crp* derivative, using the intranasal mouse model [30]. Note that the outcome of this experiment is difficult to predict since the effects of CRP on pathogenicity likely go far beyond the control of toxin expression. However, it is reasonable to assume that ETEC virulence should differ in cells lacking *crp*. The median survival of mice challenged with wild type ETEC was 53 hours and the mortality rate was 100%. Conversely, the median survival of mice challenged with Δ*crp* ETEC was 72 h and 20% of the mice survived (Fig. 9D). Thus, whilst the full extent to which CRP co-ordinates the ETEC virulence programme remains to be determined, CRP is clearly central to the pathogenic response.

## Discussion

### A complex hierarchy of salt and glucose-dependent regulation controls toxin expression

**Figure 10:**
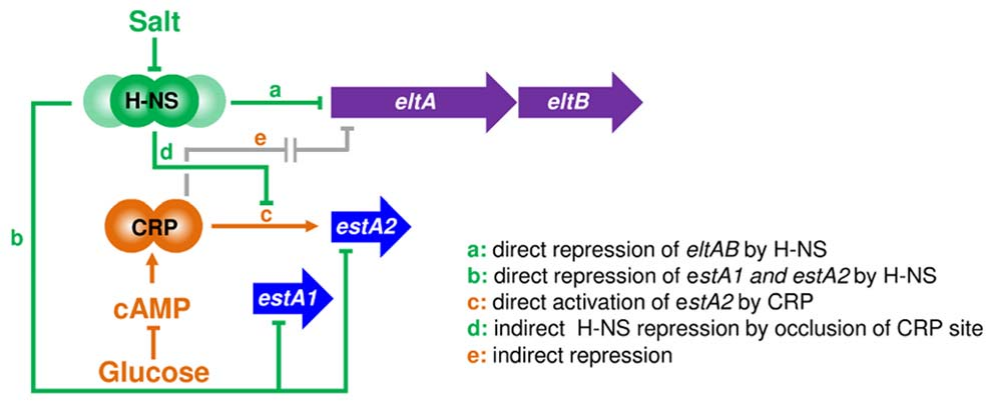
An osmo-metabolic gene regulatory circuit comprised of CRP and H-NS controls expression of LT and ST. The diagram illustrates the regulatory effects of salt, cAMP and glucose on transcription from the various ST and LT promoter regions.

We propose that toxin expression in ETEC can be controlled by osmo-metabolic flux. This is relevant to conditions in the small intestine (osmolarity equivalent to 300 mM NaCl) disease symptoms (the extrusion of cations and cAMP into the gut lumen) and treatment (the ingestion of solutions containing glucose and salt) [7]–[11], [32]. A molecular model, describing how the different signals are integrated, is illustrated in Fig. 10. Two gene regulatory proteins, CRP and H-NS, are central to our model. Hence, H-NS directly represses the expression of *eltAB*, *estA1* and *estA2* (pathways “a” and “b” in Fig. 10). For *estA2* and *eltAB* this repression can be relieved, in an H-NS dependent manner, by increased osmolarity. At P*estA2* CRP directly activates transcription by a Class I mechanism (pathway “c”). H-NS can interfere with this process by competing with CRP for binding at P*estA2* (pathway “d”). Finally, CRP can indirectly repress expression of *eltAB* via an unknown pathway that is influenced by H-NS (“e”). Both pathways “c” and “e” are sensitive to glucose availability because of their dependence on CRP. We speculate that pathway “e” may include H-NS since the effects of salt and sugar on *eltAB* expression were epistatic (Fig. 8). Our model for H-NS repression of *eltAB* is consistent with previous work [26]. However, our conclusion that *eltAB* is indirectly repressed by CRP disagrees with a previous study [27]. Even so, we were able to faithfully reproduce most of the observations previously described by Bodero and Munson [27]. We note that Bodero and Munson previously suggested that CRP may bind targets at P*eltAB* with a 7, rather than 6, base pair spacer between the two CRP half sites. Such CRP targets have never been described amongst hundreds of known CRP regulated promoters. Furthermore, we found no such CRP sites in our ChIP-seq analysis. Given that these DNA sequences can be deleted, without negating the effect of CRP on P*eltAB* activity, the regulatory effect of CRP must be indirect.

### Oral Rehydration Therapy is likely to impact on toxin expression

Our model for regulation of ST and LT expression is pertinent to both ETEC mediated disease and its treatment. ST and LT trigger the extrusion of H_2_O, cations, and cAMP (the cofactor for CRP) from the small intestine into the gut lumen [4]–[9]. Furthermore, solutions of salt and glucose are consumed by patients to reverse this process [10], [11]. We speculate that, during infection, extrusion of electrolytes and cAMP into the gut lumen could create a positive feedback loop to drive toxin expression. Importantly, our model also suggests that ORT may provide benefits beyond stimulating rehydration of the patient. The concentration of glucose used in ORT is ∼10-fold higher than is required to repress *estA2* expression. Hence, even if 90% of glucose present in ORT solutions is absorbed before reaching the site of infection, sufficient glucose should be present to down regulate toxin expression. Furthermore, even though salt is able to induce expression of *estA2* and *eltAB*, the effect is only observed at concentrations far higher than those found in ORT solutions.

### Differential regulation of *estA1* and *estA2* by CRP

Our observation that *estA1* and *estA2* are oppositely regulated by CRP is intriguing given the similarities between the promoter sequences of these genes. Differential regulation is dependent on the promoter -35 element (Fig. 5). At Class I CRP regulated promoters an αCTD protomer sits between CRP and domain 4 of the RNA polymerase σ subunit, which is bound to the promoter -35 element [12]. Thus, one possible explanation is that changes in the -35 element result in subtle repositioning of σ. This could result in unproductive interactions between αCTD and σ when CRP is present.

### H-NS prevents CRP regulation of select target genes

Our data indicate that several strong CRP binding sites in the H10407 genome are occluded by H-NS. This strongly suggests that the CRP regulon has evolved to incorporate additional environmental signals through the action of H-NS. The repressive effect of H-NS on transcription has been widely described [23]. H-NS represses transcription predominantly by occluding the binding of RNAP or by trapping RNAP at promoters [20]. Recently, it was shown that H-NS occludes many binding sites for the CRP homologue, FNR, in *E. coli* [21]. Thus, occlusion of transcription factor binding sites appears to be a major function of H-NS, especially for CRP family proteins. Note that, in order to exclude CRP from target promoters, sites of H-NS nucleation and CRP binding need not overlap precisely. For example, at both *estA1* and *estA2*, maximal H-NS binding is observed within the coding sequence of the gene (Fig. 2C). Despite this, H-NS oligomerisation across adjacent DNA is sufficient to prevent CRP binding.

### Conclusions

In summary, our model provides a framework for better understanding ETEC mediated disease and its treatment. Moreover, our catalogue of CRP and H-NS binding targets provide a useful community resource for further studies of all *E. coli* strains. In particular, our ChIP-seq data for CRP report >50 targets not identified previously in *E. coli* K-12 and 8 ETEC-specific targets. Finally, our data show how very small changes in the organisation of gene regulatory regions can have major effects on gene expression, such that transcription responds differently to the same environmental cues.

## Materials and Methods

### Strains, plasmids and oligonucleotides

ETEC strain H10407 is described by Crossman *et al.* [1]. The C-terminal *crp*-3×FLAG tag was introduced into the H10407 chromosome using the recombineering method of Stringer *et al*. [33]. Wild type *E. coli* K-12 strains JCB387 and M182 have been described previously [34], [35]. The *Δhns* M182 derivative was generated by P1 transduction of *hns*::kan from *E. coli* K12 derivative YN3144 (a gift from Ding Jin). Plasmids pRW50 and pSR are described by Lodge *et al.* [36] and Kolb *et al.* [37]. More detailed descriptions of strains and plasmids, along with the sequences of oligonucleotides, are provided in S2 Table.

### ChIP-seq

Cultures were grown to mid-log phase in M9 minimal medium with 1% (w/v) fructose at 37°C. Targeted ChIP experiments (Fig. 4 and 6) were done exactly as described by Singh and Grainger [38] using P*estA2* or P*eltAB* fragments cloned in pRW50 carried in strain M182. The ChIP-seq was done as described extensively by Singh *et al.* [25] using strain H10407. Briefly, H-NS and CRP-3×FLAG were immunoprecipitated using protein A sepharose (GE Healthcare) in combination with 2 µL of anti-H-NS or 2 µl of anti-FLAG respectively. After immunoprecipitation and washing, beads were resupended in 100 µL 1× Quick Blunting Buffer (NEB) with dNTPs (as specified by the manufacturer) and 2 µL Quick Blunting Enzyme Mix, and incubated for 30 minutes at 24°C with gentle mixing. After being collected by centrifugation, the beads were again washed and the associated DNA was A-tailed by resuspension of beads in 100 µL 1× NEB buffer #2 supplemented with 2 mM dATP and 10 units of Klenow Fragment (3′→5′ exo-; NEB). Following incubation for 30 minutes at 37°C, with gentle mixing, the beads were again collected and washed. Illumina adapters (1 µl NEXTflex ChIP-seq barcoded adapters; BioO Scientific) were added to beads resuspended in 100 µL 1× Quick Ligation reaction buffer and 4 µL Quick T4 DNA Ligase (NEB), and incubated for 15 minutes at 24°C with gentle mixing. After washing the beads, the DNA was the eluted into a fresh tube by addition of 100 µL ChIP elution buffer (50 mM Tris–HCl, pH 7.5, 10 mM EDTA, 1% SDS) and incubation at 65°C for 10 minutes. The eluate was collected by centrifugation for one minute at 4000 rpm. Crosslinks were reversed by incubation for 10 minutes at 100°C. Samples were purified by phenol extraction and precipitated with ethanol, 40 µg glycogen and 8.3 mM sodium acetate. DNA was pelleted for 15 minutes at 4°C at top speed in a microcentrifuge, washed with 70% ethanol, dried and resuspended in 11 µL H_2_O. After quantification by PCR each library was amplified, purified and resuspended in 20 µL H_2_O. Libraries were the sequenced using a HiSeq 2000 sequencer (Illumina; University at Buffalo Next Generation Sequencing Core Facility). Sequence reads were aligned to non-repetitive sequences in the *E. coli* H10407 genome using CLC Genomics Workbench and overall coverage was determined using custom Python scripts. Sequence reads have been submitted to the EBI ArrayExpress database and can be accessed using accession number E-MTAB-2917.

### Bioinformatics

ChIP-seq peaks were identified as described previously [25]. We refer to these peaks as “high stringency” peaks. A second round of peak calling was performed in which the sequence read threshold values (i.e. the minimum number of sequence reads at a given genomic position that is required for a peak to be called) was reduced by 20%. We refer to these peaks as “low stringency” peaks. MEME [39] was used to identify enriched sequence motifs in the sequences from 50 bp upstream to 50 bp downstream of the high stringency peak centres. Thus, we identified a motif closely resembling the known CRP consensus site in many of the regions surrounding high stringency ChIP-seq peaks. These CRP site sequences are included in Table 1. Those high stringency peaks for which MEME did not identify a motif were used for a second round of analysis using MEME. This also identified a motif closely resembling the known CRP consensus site. These CRP site sequences are also included in Table 1. We used MEME to identify enriched sequence motifs in the low stringency peak list. This also identified a motif closely resembling the known CRP consensus site. These CRP site sequences are also included in Table 1. “High-confidence” ChIP-seq peaks listed in Table 1 include all the high stringency peaks but only those low stringency peaks for which we identified a motif using MEME. A complete list of all peaks, including low stringency peaks for which a motif was not identified by MEME, is provided in S3 Table. In order to assess the location of CRP sites with respect to TSSs we used the targets listed in Table 1. For each target the predicted sequence from MEME was used in a BLAST search against the *E. coli* K-12 MG1655 genome. All but 11 CRP sites in ETEC had a single perfect match in the *E. coli* K-12 chromosome. For each perfect match the distance from the centre of the CRP site to all transcription start sites was calculated. Transcription start site coordinates are from Kim *et al.* [40] and Cho *et al.* [41]. Distances between −200 and +100 were selected and all other distances were discarded. Distances were then grouped in bins of 5 bp each and the most common distance bins were identified. Note that, because the position of the CRP site was transposed onto the *E. coli* K-12 genome, the distance between CRP sites and TSSs

The PWM describing CRP binding sites was generated using the PREDetector software package and our previous list of 68 CRP binding sites in the *E. coli* K-12 genome [15], [42]. Subsequent bioinformatic screens of plasmids p666 and p948 were done by importing the relevant genbank files into PREDetector and running a binding site search with a cut-off of 7 using settings that did not exclude CRP sites within genes. The “score” for each site predicted by PREDetector increases if a closer match to the PWM is found. To generate the chromosome and plasmid maps shown in Fig. 1 we used DNA plotter software [43].

Data shown in Fig. 9B–C were extracted from the publically available datasets of Kansal *et al.* [29] that measure changes in the ETEC E24377A transcriptome upon contact with Caco-2 intestinal epithelial cells. The data are hosted under the GEO accession code GSE40427. For each assay condition (planktonic and attached ETEC cells) we extracted the signal intensity for microarray probe sets A1527 (*crp*), UTI189_C1433 (*hns*), D4754 (*eltA*) and D4048 (*estA*). The average signal intensity was calculated and the fold change in transcription in attached compared to planctonic ETEC cells was determined for each time point. The data in Fig. 9C show a comparison of absolute signal intensities for probe sets A1527 (*crp*) and D4048 (*estA*) compared for each of the two replicates obtained at 30, 60 or 120 minutes after attachment to host cells. Signal intensities obtained after 30 minutes growth in LB medium (three replicates) are also included in this analysis.

### Proteins

The CRP and σ^70^ purification was done exactly as described previously [44], [45]. RNA polymerase core enzyme was purchased from Epicenter. RNA polymerase holoenzyme was generated by incubating the core enzyme with an equimolar concentration of σ^70^ at room temperature for 20 minutes prior to use. H-NS was overexpressed in T7 express cells from plasmid pJ414*hns*. After overexpressing H-NS, cells were collected from the culture by centrifugation and resuspended in buffer A (20 mM Tris-HCl pH 7.2, 1 mM EDTA and 10% (*v/v*) glycerol) containing 100 mg/ml PMSF. Cells were lysed by sonication and the sample was cleared by centrifugation. The supernatent was loaded directly onto a Heparin column (Amersham) pre-equilibrated with buffer A. A linear NaCl gradient was applied and H-NS was found to elute at approximately 500 mM NaCl. The peak fractions were pooled and diluted 3-fold with buffer A. The sample was then loaded onto an S-FF column (Amersham) pre-equilibrated with Buffer A. A NaCl gradient was applied and H-NS eluted at approximately 550 mM NaCl. The H-NS containing fractions were then dialysed against a buffer containing 20 mM Tris HCl (pH 7.2), 300 mM KCl and 10% Glycerol (*v/v*)for storage at −80°C.

### DNAse I footprinting and Electrophoretic Mobility Shift Assays

DNA fragments for DNAse I footprinting or EMSA assays were excised from pSR by sequential digestion with *Hin*dIII and then *Aat*II. After digestion, fragments were labelled at the *Hin*dIII end using [γ-^32^P]-ATP and T4 polynucleotide kinase. DNAse I footprints and EMSA experiments were then done as described by Grainger *et al.* [45] except that cAMP was added to reactions at a concentration of 0.2 mM. Radio-labelled DNA fragments were used at a final concentration of ∼10 nM. Note that all *in vitro* DNA binding reactions contained a vast excess (12.5 µg ml^−1^) of Herring sperm DNA as a non-specific competitor. Footprints were analysed on a 6% DNA sequencing gel (molecular dynamics). The results of all footprints and EMSA experiments were visualized using a Fuji phosphor screen and Bio-Rad Molecular Imager FX.

### Primer extension assays

Transcript start sites were mapped by primer extension, as described in Lloyd *et al.* [46] using RNA purified from strains carrying the 92 bp P*estA1* or 93 bp P*estA2* fragment cloned in pRW50. The 5′ end-labelled primer D49724, which anneals downstream of the *Hin*dIII site in pRW50, was used in all experiments. Primer extension products were analysed on denaturing 6% polyacrylamide gels, calibrated with size standards, and visualized using a Fuji phosphor screen and Bio-Rad Molecular Imager FX.

### *In vitro* transcription assays

The *in vitro* transcription experiments were performed as described previously Savery *et al.* [35] using the system of Kolb *et al.* [38]. A Qiagen maxiprep kit was used to purify supercoiled pSR plasmid carrying the different promoter inserts. This template (∼16 µg ml−1) was pre-incubated with purified CRP in buffer containing 0.2 mM cAMP, 20 mM Tris pH 7.9, 5 mM MgCl_2_, 500 µM DTT, 50 mM KCl, 100 µg ml^−1^ BSA, 200 µM ATP, 200 µM GTP, 200 µM CTP, 10 µM UTP with 5 µCi [α-32P]-UTP. The reaction was started by adding purified *E. coli* RNA polymerase. Labelled RNA products were analysed on a denaturing polyacrylamide gel.

### β-galactosidase assays and addition of glucose and salt to growth medium

β-Galactosidase assays were done using the protocol of Miller [47]. All assay values are the mean of three independent experiments with a standard deviation <10% of the mean. Cells were grown aerobically at 37°C to mid-log phase in LB medium unless stated otherwise. For all experiments investigating the effects of glucose and salt M9 minimal medium was used so that the glucose and salt concentrations could be controlled more accurately. The amount of glucose is shown as percentage *w/v*. The addition of “salt” refers to a 3∶1 molar ration of NaCl to KCl. We have arbitrarily described 30 mM NaCl and 10 mM KCl as being a “1%” salt solution.

### Intranasal mouse infection model assays

Strains of ETEC were grown in Luria Broth (LB) to an OD_600_ of 1.0. Groups of 10 mice (8–10 week old BALB/c) were infected intranasally with approximately 1×10^9^ colony forming units of bacteria in 100 µl of inoculums according to Byrd *et al.* [30]. Mice were monitored daily for 6 days post-infection for weight and morbidity.

### Ethics statement

The protocol 12-02-015IBT “Oral Immunization of Mice with Enterotoxigenic: *E coli* (ETEC)” has been approved by the Noble Life Sciences IACUC committee. All animal care and use procedures adhere to the guidelines set by the Public Health Service Policy, U.S. Dept. of Agriculture (USDA) and the Guide for the Care and Use of Laboratory Animals of the National Institutes of Health.

## Supporting Information

S1 Fig **Binding of CRP to predicted targets** ***in vitro*** **.** A) The data show binding of CRP to a target from each of the bins shown in Fig. 2. B) CRP binding to remaining targets scoring >10. CRP was used at concentrations of 0, 175, 350 or 700 nM. The “score” describes how well the predicted target matches the PWM.
(PDF)

S2 Fig **Promoter DNA fragments used in this work.** A) ETEC H10407 P*estA2* containing DNA fragments. B) ETEC H10407 derived DNA fragments containing P*estA1*. C) ETEC H10407 P*eltAB* containing sequences. D) DNA fragments containing sequences upstream of the *estA2* and *eltAB* genes of ETEC E24377A.
(PDF)

S3 Fig **A) Mutations in P** ***estA2*** **UP-element alter the migration of the promoter DNA on an agarose gel.** The DNA sequences used are shown in part (i) and the mobility of the fragments, on an agarose gel, are shown in part (ii). Note that each sample has been loaded in duplicate. B) Mutating the UP-element renders P*estA2* uninducible by CRP. Part (i) shows LacZ activity data for the different promoter fragments cloned in pRW50. The pRW50 derivatives were used to transform M182 or the Δ*crp* derivative. Part (ii) shows the result of in vitro transcription assays using the different promoter fragments, cloned in pSR, as a template.
(PDF)

S4 Fig **Activity of different promoter::** ***lacZ*** **fusions in the presence of increasing glucose and salt concentrations.** The figure shows β-galactosidase activity measurements for lysates obtained from cultures of M182 carrying the A) *estA1* B) *estA2* or C) *eltAB* promoters cloned in pRW50. Panel D) shows β-galactosidase activity values for lysates of M182 and M182Δ*hns* cells, carrying the *estA2* promoter, or a derivative lacking the CRP site, cloned in pRW50. Cells were grown in the presence or absence of 2% glucose. Assays were done in M9 minimal medium so that the glucose and salt concentrations could be more accurately controlled.
(PDF)

S1 Table **Putative CRP binding targets on ETEC plasmids p948 and p666 identified by PREDetector.**
(DOCX)

S2 Table **Strains, plasmids and oligonucleotides.**
(DOCX)

S3 Table **All CRP binding sites on the ETEC H10407 chromosome identified by ChIP-seq.**
(DOCX)
