# Supplementary figures and images for: The Molecular Basis for Control of ETEC Enterotoxin Expression in Response to Environment and Host
Source: PLoS Pathog. 2015 Jan 8;11(1):e1004605. doi: 10.1371/journal.ppat.1004605 (PMC4287617; doi:10.1371/journal.ppat.1004605)

Figure S1

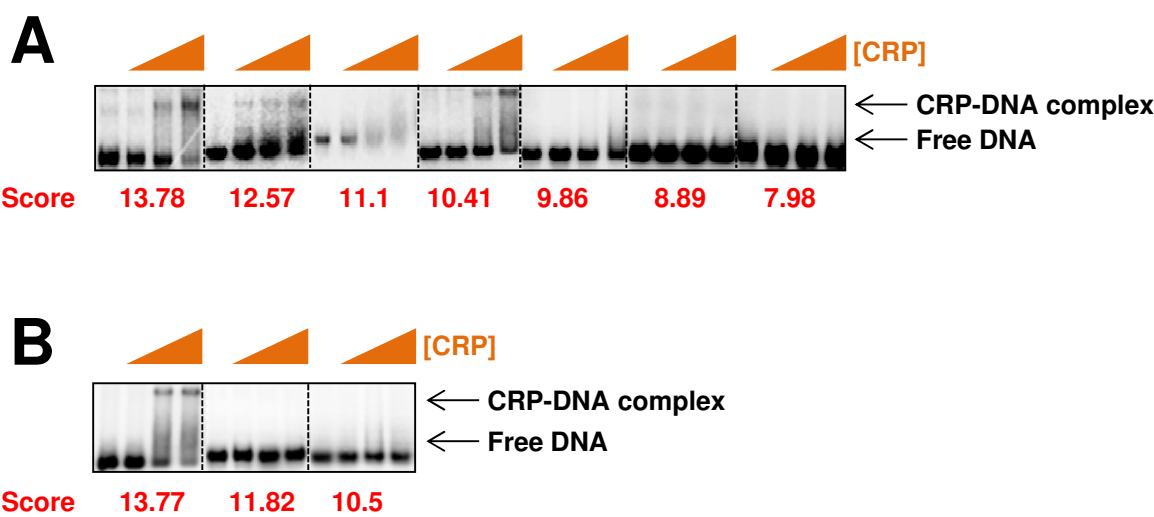

Supplement: S1 Fig — Binding of CRP to predicted targets in vitro . A) The data show binding of CRP to a target from each of the bins shown in Fig. 2. B) CRP binding to remaining targets scoring >10. CRP was used at concentrations of 0, 175, 350 or 700 nM. The “score” describes how well the predicted target matches the PWM. (PDF) [file ppat.1004605.s001.pdf]
