# Supplementary material for: The Molecular Basis for Control of ETEC Enterotoxin Expression in Response to Environment and Host
Source: PLoS Pathog. 2015 Jan 8;11(1):e1004605. doi: 10.1371/journal.ppat.1004605 (PMC4287617; doi:10.1371/journal.ppat.1004605)
Supplement: S2 Fig — Promoter DNA fragments used in this work. A) ETEC H10407 PestA2 containing DNA fragments. B) ETEC H10407 derived DNA fragments containing PestA1. C) ETEC H10407 PeltAB containing sequences. D) DNA fragments containing sequences upstream of the estA2 and eltAB genes of ETEC E24377A. (PDF) [file ppat.1004605.s002.pdf]

Figure S2

***estA* coding sequence**
***eltAB* coding sequence**
transcription start site
CRP site predicted by Bodero and Munson
CRP binding site identified in this work
UP element
Promoter -10 and -35 elements

A

P*estA2* 93 base pair fragment

TATGATACACA**TCACAAAAAAATAAAAAA**GT**TTGCGC**AATCGTTCTGATTTTGAT**TTAAAT**ATTCGTG
GACGACGTGTTTCGGAGGTAAT**ATG**

P*estA2* 93 base pair fragment with UP element mutated

TATGATACACA**TCACAA**gc**A**c**A**gcg**A**c**AA**GT**TTGCGC**AATCGTTCTGATTTTGAT**TTAAAT**ATTCGTG
GACGACGTGTTTCGGAGGTAAT**ATG**

P*estA2* 460 base pair fragment

CCGGGGCGGTTCATTGTTATTTTTTTTGTGATTAACCCCCACAAAAATAGTCATTCAACATAAATATA
TCATTTTATTTAAGCAATCTTTTCTGTTTTTTCGGCCGCCCTAAAACATAATATTATTATGCTCTTCG
TAGCGGAGAGTATAG**TATGA**TACACA**TCACAAAAAAATAAAAAA**GT**TTGCGC**AATCGTTCTGATTTTG
AT**TTAAAT**ATTCGTGGACGACGTGTTTCGGAGGTAAT**ATGAAAAAATCAATATTATTTATTTTTCTTT**
CTGTATTATCTTTTTCACCTTTCGCTCAGGATGCTAAACCAGCAGGGTCTTCAAAAGAAAAAATTACA
CTAGAATCGAAAAAATGTAACATTGTAAAAAAAAATAATGAAAGTAGTCCTGAAAGCATGAATAGTAG
CAATTACTGCTGTGAATTGTGTTGTAATCCTGCTTGTACCGGGTGCTATTAA

P*estA2* 460 base pair fragment with CRP site mutated

CCGGGGCGGTTCATTGTTATTTTTTTTGTGATTAACCCCCACAAAAATAGTCATTCAACATAAATATA
TCATTTTATTTAAGCAATCTTTTCTGTTTTTTCGGCCGCCCTAAAACATAATATTATTATGCTCTTCG
TAGCGGAGAGTATAGAAA**G**TTACACAA**C**T**C**T**AAAAAATAAAAAA**GT**TTGCGC**AATCGTTCTGATTTTG
AT**TTAAAT**ATTCGTGGACGACGTGTTTCGGAGGTAAT**ATGAAAAAATCAATATTATTTATTTTTCTTT**
CTGTATTATCTTTTTCACCTTTCGCTCAGGATGCTAAACCAGCAGGGTCTTCAAAAGAAAAAATTACA
CTAGAATCGAAAAAATGTAACATTGTAAAAAAAAATAATGAAAGTAGTCCTGAAAGCATGAATAGTAG
CAATTACTGCTGTGAATTGTGTTGTAATCCTGCTTGTACCGGGTGCTATTAA

B

P*estA1* 92 base pair fragment

CATGATGCAAC**TCACAAAAAAAATAAAAAAATTGCAA**AATCCGTTTAACTAATC**TCAAAT**ATCCGTGA
AACAACATGACGGGAGGTAACATG

P*estA1* 350 base pair fragment

ATGAAAATAATATATAAAAAGCGAGTGTACCTCGACATATAACATGATGCAACTCACAAAAAAAATAA
AAAAATTGCAAAATCCGTTTAACTAATCTCAAATATCCGTGAAACAACATGACGGGAGGTAACATGAA
AAAGCTAATGTTGGCAATTTTTATTTCTGTATTATCTTTCCCCTCTTTTAGTCAGTCAACTGAATCAC
TTGACTCTTCAAAAGAGAAAATTACATTAGAGACTAAAAAGTGTGATGTTGTAAAAAACAACAGTGAA
AAAAAATCAGAAAATATGAACAACACATTTTACTGCTGTGAACTTTGTTGTAATCCTGCCTGTGCTGG
ATGTTATTAA

C

P*eltAB* 1127 base pair fragment

TTCTGGTGTGGACTTTCTGGTGCTCCAGGTTGTGTGACATGGGAACTCATTCTGGATGGTTACTCTGA
AAGCTCATATTCTGCCACCCCCCGATTTGCAGCCGCCAAGCTGCCGTGGTTCAAGTCGCGACTAATAA
AAATAATCAGGTTGCCATGATTCAATGTACACCTTTCTCACATTCGTCTCCGGCATGAAAACGATGCA
CTCTTTCTTTATCGCTTTCACTACACATTTTATCCTCGCATGGATGTTTTATAAAAAACATGATTGAC
ATCATGTTGCATATAGGTTAAACAAAACAAGTGGCGTTATCTTTTTCCGGATTGTCTTCTTGTATGAT
ATATAAGTTTTCCTCGATGAAAAATATAACTTTCATTTTTTTTATTTTATTAGCATCGCCATTATATG
CAAATGGCGACAAATTATACCGTGCTGACTCTAGACCCCCAGATGAAATAAAACGTTCCGGAGGTCTT
ATGCCCAGAGGGCATAATGAGTACTTCGATAGAGGAACTCAAATGAATATTAATCTTTATGATCACGC
GAGAGGAACACAAACCGGCTTTGTCAGATATGATGACGGATATGTTTCCACTTCTCTTAGTTTGAGAA
GTGCTCACTTAGCAGGACAGTCTATATTATCAGGATATTCCACTTACTATATATATGTTATAGCGACA
GCACCAAATATGTTTAATGTTAATGATGTATTAGGCGTATACAGCCCTCACCCATATGAACAGGAGGT
TTCTGCGTTAGGTGGAATACCATATTCTCAGATATATGGATGGTATCGTGTTAATTTTGGTGTGATTG
ATGAACGATTACATCGTAACAGGGAATATAGAGACCGGTATTACAGAAATCTGAATATAGCTCCGGCA
GAGGATGGTTACAGATTAGCAGGTTTCCCACCGGATCACCAGGCTTGGAGAGAAGAACCCTGGATTCA
TCATGCACCACAAGGTTGTGGAAATTCATCAAGAACAATTACAGGTGATACTTGTAATGAGGAGACCC
AGAATCTGAGCACAATATATCTCAGGAAATATCAATCAAAAGTTAAGAGGCAGATATTTTCAGACTAT
CAGTCAGAGGTTGACATATATAACAGGATTCGGAATGAA

P*eltAB* 359 base pair fragment

TTCTGGTGTGGACTTTCTGGTGCTCCAGGTTGTGTGACATGGGAACTCATTCTGGATGGTTACTCTGA
AAGCTCATATTCTGCCACCCCCCGATTTGCAGCCGCCAAGCTGCCGTGGTTCAAGTCGCGACTAATAA
AAATAATCAGGTTGCCATGATTCAATGTACACCTTTCTCACATTCGTCTCCGGCATGAAAACGATGCA
CTCTTTCTTTATCGCTTTCACTACACATTTTATCCTCGCATGGATGTTTTATAAAAAACATGATTGAC
ATCATGTTGCATATAGGTTAAACAAAACAAGTGGCGTTATCTTTTTCCGGATTGTCTTCTTGTATGAT
ATATAAGTTTTCCTCGATG

P*eltAB* 118 base pair fragment

GCATGGATGTTTTATAAAAAACATGATTGACATCATGTTGCATATAGGTTAAACAAAACAAGTGGCGT
TATCTTTTTCCGGATTGTCTTCTTGTATGATATATAAGTTTTCCTCGATG

P*eltAB* 118 base pair fragment with putative CRP site mutated

GCATGGATGTTTTATAAAAAACAACATTGACATGTTGTTGCATATAGGTTAAACAAAACAAGTGGCGT
TATCTTTTTCCGGATTGTCTTCTTGTATGATATATAAGTTTTCCTCGATG

D

P*eltAB* 1126 base pair fragment from ETEC E24377A

TTCTGGTGTGGACTTTCTGGTGCTCCAGGTTGTGTGACATGGGAACTCATTCTGGATGGTTACTCTGA
AAGCTCATATTCTGCCCCCCCCGATTTGCAGCCGCCAGGCTGCCGTGGTTCAAGTCGCGACTAATAAA
AATAATCAGGTTGCCATGATTCAATGTACACCTTTCTCACATTCGTCTCCGGCATGAAAACGATGCAC
TCTTTCTTTATCGCTTTCACTACACATTTTATCCTCGCATGGATGTTTTATAAAAAACATGA**TTGACA**
TCATGTTGCATATAGG**TTAAAC**AAAACAAGTAGCGTTATCTTTTTCCGGATTGTCTTCTTGTATGATA
TATAAGTTTTCCTCGATGAAAAATATAACTTTCATTTTTTTTATTTTATTAGCATCGCCATTATATGC
AAATGGCGACAAATTATACCGTGCTGACTCTAGACCCCCAGATGAAATAAAACGTTCCGGAGGTCTTA
TGCCCAGAGGGCATAATGAGTACTTCGATAGAGGAACTCAAATGAATATTAATCTTTATGATCACGCG
AGAGGAACACAAACCGGCTTTGTCAGATATGATGACGGATATGTTTCCACTTCTCTTAGTTTGAGAAG
TGCTCACTTAGCAGGACAGTCTATATTATCAGGATATTCCACTTACTATATATATGTTATAGCGACAG
CACCAAATATGTTTAATGTTAATGATGTATTAGGCGTATACAGCCCTCACCCATATGAACAGGAGGTT
TCTGCGTTAGGTGGAATACCATATTCTCAGATATATGGATGGTATCGTGTTAATTTTGGTGTGATTGA
TGAACGATTACATCGTAACAGGGAATATAGAGACCGGTATTACAGAAATCTGAATATAGCTCCGGCAG
AGGATGGTTACAGATTAGCAGGTTTCCCACCGGATCACCAAGCTTGGAGAGAAGAACCCTGGATTCAT
CATGCACCACAAGGTTGTGGAAATTCATCAAGAACAATTACAGGTGATACTTGTAATGAGGAGACCCA
GAATCTGAGCACAATATATCTCAGGAAATATCAATCAAAAGTTAAGAGGCAGATATTTTCAGACTATC
AGTCAGAGGTTGACATATATAACAGAATTCGGGATGAA

P*estA2* 460 base pair fragment from ETEC E24377A

CATTAAGTATGCATTGTTATTTTTTTGTGATTAACCCCACAAAAACAGTCATTAAACATAAATATATC
ATTTTGCTTAAACAATCTTTTCTGTTTTTTTCGGTCGCCGAAAAAGATAATATTACTATGCTCTTCGT
AGCGGAGAGTATAG**TATGA**TGTTCA**TCACAAAAAAATAAAAAAA**GT**TTGCGC**AATCGTTCTGGTTTTG
AT**TCAAAT**GTTCGTGGATGCCATGTTCCGGAGGTAATATGAAGAAATCAATATTATTTATTTTTCTTT
CTGTATTGTCTTTTTCACCTTTCGCTCAGGATGCTAAACCAGTAGAGTCTTCAAAAGAAAAAATCACA
CTAGAATCAAAAAAAATGTAACATTGCAAAAAAAAAGTAATAAAAGTGGTCCTGAAAGCATGAATAGTAG
CAATTACTGCTGTGAATTGTGTTGTAATCCTGCTTGTACCGGGTGCTATTAA
