# Supplementary material for: The Molecular Basis for Control of ETEC Enterotoxin Expression in Response to Environment and Host
Source: PLoS Pathog. 2015 Jan 8;11(1):e1004605. doi: 10.1371/journal.ppat.1004605 (PMC4287617; doi:10.1371/journal.ppat.1004605)

Figure S3

A

i **P*estA2* 93 base pair fragment**

TATGATACACATCACAAAAAAATAAAAAAGTTTGCGCAATCGTTCTGATTTTGATTTAAATATTCGTG
GACGACGTGTTTCGGAGGTAATATG

P*estA2* 93 base pair fragment with UP element mutated

TATGATACACATCACAAgcAcAgcgAcAAGTTTGCGCAATCGTTCTGATTTTGATTTAAATATTCGTG
GACGACGTGTTTCGGAGGTAATATG

ii

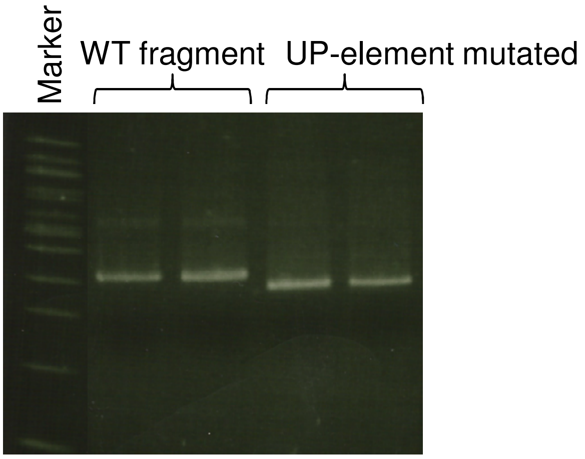

B

i

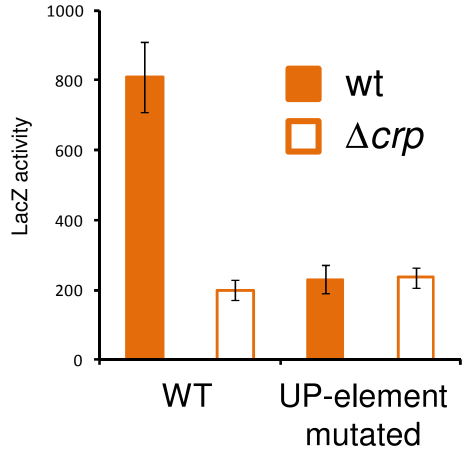

ii

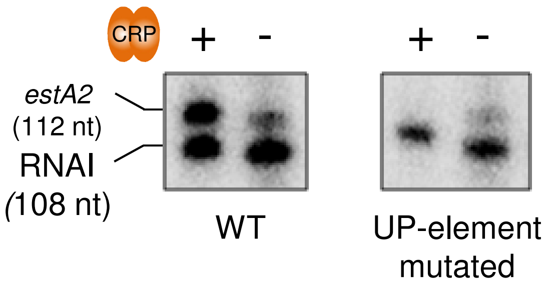

Supplement: S3 Fig — A) Mutations in P estA2 UP-element alter the migration of the promoter DNA on an agarose gel. The DNA sequences used are shown in part (i) and the mobility of the fragments, on an agarose gel, are shown in part (ii). Note that each sample has been loaded in duplicate. B) Mutating the UP-element renders PestA2 uninducible by CRP. Part (i) shows LacZ activity data for the different promoter fragments cloned in pRW50. The pRW50 derivatives were used to transform M182 or the Δcrp derivative. Part (ii) shows the result of in vitro transcription assays using the different promoter fragments, cloned in pSR, as a template. (PDF) [file ppat.1004605.s003.pdf]
