# Supplementary material for: The Molecular Basis for Control of ETEC Enterotoxin Expression in Response to Environment and Host
Source: PLoS Pathog. 2015 Jan 8;11(1):e1004605. doi: 10.1371/journal.ppat.1004605 (PMC4287617; doi:10.1371/journal.ppat.1004605)
Supplement: S4 Fig — Activity of different promoter:: lacZ fusions in the presence of increasing glucose and salt concentrations. The figure shows β-galactosidase activity measurements for lysates obtained from cultures of M182 carrying the A) estA1 B) estA2 or C) eltAB promoters cloned in pRW50. Panel D) shows β-galactosidase activity values for lysates of M182 and M182Δhns cells, carrying the estA2 promoter, or a derivative lacking the CRP site, cloned in pRW50. Cells were grown in the presence or absence of 2% glucose. Assays were done in M9 minimal medium so that the glucose and salt concentrations could be more accurately controlled. (PDF) [file ppat.1004605.s004.pdf]

# Figure S4

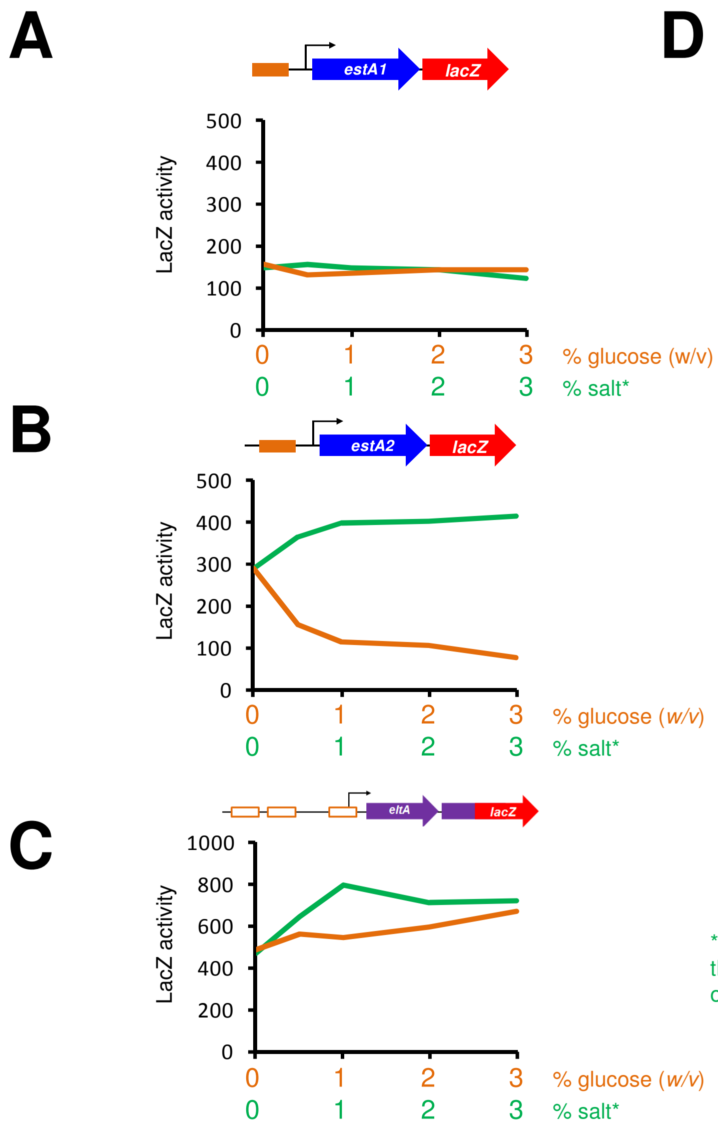


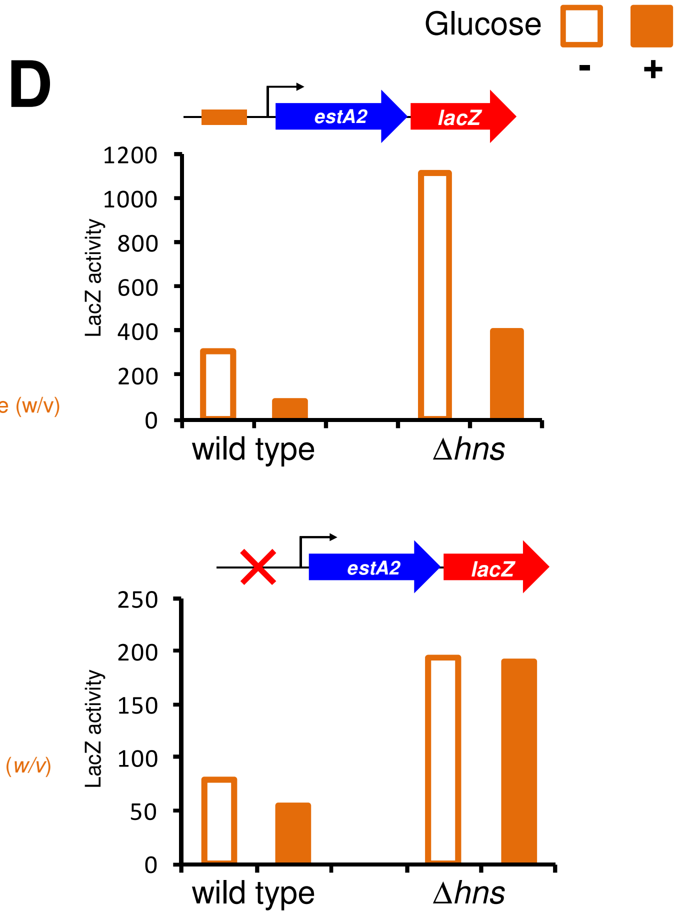


*NaCl and KCl were added together in a 3:1 molar ratio. In these experiments, 1 % salt describes a final concentration of 30 mM NaCl and 10 mM KCl.
