# Supplementary material for: The Molecular Basis for Control of ETEC Enterotoxin Expression in Response to Environment and Host
Source: PLoS Pathog. 2015 Jan 8;11(1):e1004605. doi: 10.1371/journal.ppat.1004605 (PMC4287617; doi:10.1371/journal.ppat.1004605)
Supplement: S1 Table — Putative CRP binding targets on ETEC plasmids p948 and p666 identified by PREDetector. (DOCX) [file ppat.1004605.s005.docx]

**Table S1: Putative CRP binding targets on ETEC plasmids p948 and p666 identified by PREDetector**

| **Bin^a^** | **Predicted CRP site^b^** | **SCORE^c^** | **TARGET^d^** | **Bound *in vitro* (n.d. = not done)^e^** |
| --- | --- | --- | --- | --- |
| 13 to 14 | ttt**tgtga**aattaa**tcaca**aaa | 13.78 | ETEC_p666_0090 | Yes |
|  | taaa**gtga**taaaaa**tcaca**taa | 13.77 | ETEC_p948_0870c, aatC | Yes |
| 12 to 13 | ttt**tgtga**tgtgta**tcata**cta | 12.57 | ETEC_p948_0360c, estA2 | Yes |
| 11 to 12 | ttt**t**a**tga**aatcaa**tcaca**aaa | 11.82 | ETEC_p666_0110 | No |
|  | ata**t**t**tga**acgaag**tca**a**a**ttt | 11.1 | ETEC_p666_0360, traJ | Yes |
| 10 to 11 | taa**tgt**a**a**aataaa**t**t**a**a**a**ata | 10.5 | ETEC_p948_0510 | No |
|  | ttt**tgtga**gttgca**tca**tgtta | 10.41 | ETEC_p666_0750, estA1 | Yes |
| 9 to 10 | ata**tgtga**tattaa**t**ag**ca**caa | 9.86 | ETEC_p948_0410, cfaC | No |
|  | tta**t**t**tga**agcaaa**tca**acttt | 9.85 | ETEC_p948_0490, traJ | n.d. |
|  | taa**tgtga**ttttga**t**a**a**tgaaa | 9.62 | ETEC_p948_0230c, repA2 | n.d. |
|  | aac**t**t**t**a**a**taaacac**caca**tta | 9.56 | ETEC_p948_0830 | n.d. |
|  | att**t**t**tga**tatgtc**t**g**aca**tta | 9.53 | ETEC_p948_0700 | n.d. |
|  | att**t**t**t**t**a**tatata**t**tg**ca**tta | 9.5 | ETEC_p948_0700 | n.d. |
|  | aat**t**a**tga**tgttgta**ca**t**a**taa | 9.36 | ETEC_p58_0005 | n.d. |
|  | aag**tg**c**ga**atctat**t**a**aca**ata | 9.08 | ETEC_p948_0800 | n.d. |
|  | aaa**t**aa**ga**tacaca**t**a**a**a**a**aaa | 9 | ETEC_p52_0001 | n.d. |
|  | att**tgtga**atcact**tcac**gacc | 9 | ETEC_p52_0006, rop | n.d. |
| 8 to 9 | ttt**tgtg**gagtggg**t**t**a**a**a**tta | 8.89 | ETEC_p948_0510 | No |
|  | aaac**gt**t**a**ctacgt**tcac**gttt | 8.73 | ETEC_p666_0880, relE | n.d. |
|  | aaaat**tg**taataga**t**a**a**a**a**aaa | 8.63 | ETEC_p948_0910, cexE | n.d. |
|  | atag**gt**a**a**atctgt**tca**a**a**aaa | 8.62 | ETEC_p948_0790 | n.d. |
|  | tgca**gt**a**a**ttaacg**tcaca**ttt | 8.59 | ETEC_p948_0020, eatA | n.d. |
|  | aaa**tg**c**ga**tgaagc**t**a**a**t**a**aat | 8.46 | ETEC_p666_0560, stbB | n.d. |
|  | aaac**g**g**ga**agctaa**tc**g**ca**taa | 8.43 | ETEC_p948_0530 | n.d. |
|  | aatct**tga**aaaata**t**a**ac**caaa | 8.28 | ETEC_p948_0870 | n.d. |
|  | tac**tgtga**attaat**t**a**ac**gttg | 8.28 | ETEC_p948_0120, etpB | n.d. |
|  | aat**tgtg**gaagaaa**t**t**aca**atg | 8.25 | 40522 -> 40543 | n.d. |
|  | ttt**t**act**a**tcaata**tca**t**a**tta | 8.24 | ETEC_p666_0750, sta1 | n.d. |
|  | ataat**tga**tataca**t**a**a**actta | 8.16 | ETEC_p948_0990 | n.d. |
|  | ttt**t**c**tga**atgaga**tc**g**c**cttt | 8.14 | ETEC_p58_0001 | n.d. |
|  | caa**tgt**t**a**tttata**tca**ttaaa | 8.12 | ETEC_p948_0990 | n.d. |
|  | taa**t**a**t**c**a**aataaag**caca**act | 8.08 | ETEC_p948_0120, etpB | n.d. |
|  | aaa**tgt**attccgtc**tcaca**tgt | 8.04 | ETEC_p948_0700 | n.d. |
|  | ttt**tgtga**ttttcta**c**tatatt | 8.04 | ETEC_p948_0890c | n.d. |
| 7 to 8 | att**t**t**t**t**a**acaact**t**t**a**t**a**ttt | 7.98 | ETEC_p948_0890c | No |
|  | aaa**t**t**t**t**a**tctaaaga**a**a**a**taa | 7.9 | 42550 -> 42571 | n.d. |
|  | aaagaca**a**cataaa**t**a**aca**ttt | 7.9 | ETEC_p948_0020c, eatA | n.d. |
|  | aatc**gtga**cgcaag**t**t**ac**gaaa | 7.85 | ETEC_p948_0500c, traM | n.d. |
|  | ttacact**a**taaaca**tcaca**gtt | 7.83 | 42327 -> 42348 | n.d. |
|  | taa**t**a**t**ttagaaca**tca**t**a**ata | 7.82 | 44227 -> 44248 | n.d. |
|  | caaat**tga**accaga**tca**a**a**atc | 7.77 | ETEC_p948_0490c, traJ | n.d. |
|  | att**t**c**tga**tacattaa**ac**gtat | 7.65 | 43228 -> 43249 | n.d. |
|  | aaa**tg**aattttcaa**tca**a**a**tta | 7.65 | ETEC_p666_0870c | n.d. |
|  | ttt**t**a**t**t**a**ttccata**caca**taa | 7.64 | ETEC_p666_0650c, eltB | n.d. |
|  | aag**tg**c**ga**atctat**t**a**aca**ata | 7.62 | ETEC_p948_0800c | n.d. |
|  | attca**tg**cagcaaa**tcaca**tca | 7.61 | ETEC_p948_0500c, traM | n.d. |
|  | aaa**tgt**t**a**tatcttc**c**t**c**ttta | 7.58 | ETEC_p666_0350c, traY | n.d. |
|  | tta**tgt**t**a**actcaa**t**tt**ca**ata | 7.52 | 37232 -> 37253 | n.d. |
|  | aaactca**a**attgag**tcaca**aca | 7.51 | ETEC_p948_0680, stbA | n.d. |
|  | aat**t**a**tg**gtgataa**t**a**a**t**a**ttt | 7.48 | 41344 -> 41365 | n.d. |
|  | aataa**t**a**a**taaaaag**ca**a**a**aaa | 7.47 | ETEC_p948_1070c | n.d. |
|  | aaa**tgt**a**a**ttgataat**a**a**a**aaa | 7.45 | 38188 -> 38209 | n.d. |
|  | aat**tgtg**gaagaaa**t**t**aca**atg | 7.44 | 40522 -> 40543 | n.d. |
|  | atc**t**tc**ga**ccatat**tc**g**ca**tat | 7.4 | 47921 -> 47942 | n.d. |
|  | cat**tgt**c**a**tattta**tca**g**a**aaa | 7.39 | ETEC_p948_0990c | n.d. |
|  | caa**tgt**t**a**tttata**tca**ttaaa | 7.39 | ETEC_p948_0990c | n.d. |
|  | att**t**c**t**t**a**tagaaa**t**t**ac**tttt | 7.38 | ETEC_p948_0800c | n.d. |
|  | aatg**g**c**ga**tgctaa**t**a**a**a**a**taa | 7.37 | ETEC_p666_0660c, eltA | n.d. |
|  | ttt**tgt**t**a**ttatta**tc**ta**a**gct | 7.31 | ETEC_p948_0870c | n.d. |
|  | gaaca**tga**gcagca**tca**t**a**aaa | 7.31 | ETEC_p666_0360c, traJ | n.d. |
|  | tac**tg**c**g**gcgcagt**tcac**gatt | 7.3 | ETEC_p948_0050c | n.d. |
|  | aat**t**t**tg**gtctcgg**tca**g**a**tat | 7.3 | ETEC_p666_0650c, eltB | n.d. |
|  | aatc**gtga**actgcgc**c**g**ca**gta | 7.3 | ETEC_p948_0240 | n.d. |
|  | ttt**t**t**t**t**a**attgcg**t**tg**ca**tat | 7.27 | ETEC_p948_0900c | n.d. |
|  | ataa**gtga**tagtct**t**a**a**t**a**cta | 7.26 | ETEC_p948_0470 | n.d. |
|  | taa**tgtg**ttaggca**t**t**a**acatt | 7.25 | 61025 -> 61046 | n.d. |
|  | tga**tgtg**gtatgtt**t**t**a**tcttt | 7.21 | ETEC_p666_0110c | n.d. |
|  | caa**t**a**tga**tttagt**t**a**ac**ggta | 7.2 | ETEC_p666_0550, stbA | n.d. |
|  | aaac**g**ca**a**tgtatt**tca**ttatt | 7.2 | 60523 -> 60544 | n.d. |
|  | tcag**gtga**tgcact**tca**a**a**aag | 7.19 | 29938 -> 29959 | n.d. |
|  | atc**t**t**tga**taattt**tc**t**ca**atg | 7.19 | 25210 -> 25231 | n.d. |
|  | ttactca**a**tcctct**tcaca**aca | 7.18 | ETEC_p666_0560, stbB | n.d. |
|  | att**tgtg**tcatggg**t**t**ac**cata | 7.16 | 75209 -> 75230 | n.d. |
|  | ata**t**t**tga**tatctg**t**g**a**t**a**tct | 7.14 | 43102 -> 43123 | n.d. |
|  | aga**t**taa**a**aaaacac**caca**aaa | 7.14 | 90310 -> 90331 | n.d. |
|  | aatga**tga**atatca**tca**attat | 7.13 | 39580 -> 39601 | n.d. |
|  | ttt**t**tcc**a**tctgca**tca**a**a**att | 7.12 | 67889 -> 67910 | n.d. |
|  | ttt**t**a**tga**tggata**t**agt**a**cta | 7.12 | 42475 -> 42496 | n.d. |
|  | aat**tgt**t**a**ttggtgaa**a**t**a**att | 7.12 | 5162 -> 5183 | n.d. |
|  | ttg**t**t**t**t**a**tcaaaa**tca**tgttt | 7.11 | 4348 -> 4369 | n.d. |
|  | tct**tgtga**gggaga**t**tgt**a**ttt | 7.11 | 77103 -> 77124 | n.d. |
|  | ata**t**ta**ga**acgata**t**tg**ca**taa | 7.11 | 74898 -> 74919 | n.d. |
|  | tac**tgtga**attaat**t**a**ac**gttg | 7.09 | ETEC_p948_0120c, etpB | n.d. |
|  | aac**t**t**t**a**a**taaacac**caca**tta | 7.09 | ETEC_p948_0830c | n.d. |
|  | tgt**t**t**t**ttacaaca**tcaca**ctt | 7.08 | ETEC_p666_0750c, sta1 | n.d. |
|  | tgt**t**t**t**ttacaaca**tcaca**ctt | 7.08 | ETEC_p666_0750c, sta1 | n.d. |
|  | atag**gt**a**a**atctgt**tca**a**a**aaa | 7.08 | ETEC_p948_0790c | n.d. |
|  | taac**gtg**gcattaac**cac**gtaa | 7.07 | ETEC_p948_0490c, traJ | n.d. |
|  | tga**t**t**tga**ctgctc**t**t**a**a**a**ttt | 7.06 | ETEC_p666_0870c | n.d. |
|  | aaac**g**g**ga**acgggt**tcaca**aac | 7.03 | 10961 -> 10982 | n.d. |
|  | aaa**tg**ca**a**acttta**t**g**a**t**a**tat | 7.02 | 37876 -> 37897 | n.d. |
|  | aaa**tgt**attccgtc**tcaca**tgt | 7 | 61211 -> 61232 | n.d. |

^a^Predicted CRP sites were organised into bins on the basis of the site score.

^b^Sequence of the predicted CRP site. Matches to the consensus CRP binding sequence are shown in bold.

^c^The score for each predicted site assigned by PREDetector. Higher scoring sites have a closer match to the Position Weight Matrix (PWM) used to identify potential CRP binding sites.

^d^The position of predicted CRP targets with respect to adjacent genes. If the CRP binding target was between divergent genes both gene names are shown. If the target was between convergent genes the co-ordinates of the site are shown.

^e^Sites tested for CRP binding *in vitro* using electrophoretic mobility shift assays.
