# Supplementary material for: The Molecular Basis for Control of ETEC Enterotoxin Expression in Response to Environment and Host
Source: PLoS Pathog. 2015 Jan 8;11(1):e1004605. doi: 10.1371/journal.ppat.1004605 (PMC4287617; doi:10.1371/journal.ppat.1004605)
Supplement: S2 Table — Strains, plasmids and oligonucleotides. (DOCX) [file ppat.1004605.s006.docx]

**Table S2: Strains, plasmids and oligonucleotides**

| **Name** | **Description** | **Source** |
| --- | --- | --- |
| **Bacterial strains** | | |
|  |  |  |
| H10407 |  | Crossman *et al*. (2010) |
| H10407 *crp*3xFLAG | H10407 *crp*3xFLAG | This work |
| GPM1812 | H10407Δ*crp* Kan^R^ | Espert *et. al*. (2011) |
| M182 | Δ*lac* *galK* *galU* *strA* | Busby *et al*. (1983) |
| M182Δ*crp* | Δ*lac* *galK* *galU* *strA* Δ*crp* | Busby *et al*. (1983) |
| M182*Δhns* | M182*Δhns* Kan^R^ | This work |
| T7 express |  | Invitrogen |
|  |  |  |
| **Bacterial plasmids** |  |  |
| pRW50 | Broad-host-range *lac* fusion vector for cloning promoters on *Eco*RI–*Hin*dIII fragments: contains the RK2 origin of replication and encodes TcR | Lodge *et. al*. (1992) |
| pSR | pBR322-derived plasmid containing an *Eco*RI–*Hin*dIII fragment upstream of the λ*oop* transcription terminator | Kolb *et. al*. (1995) |
|  |  |  |
| pJ414*hns* | A high copy number plasmid encoding *hns* under the control of an inducible T7 promoter. Also encodes Amp^R^. | This work |
|  |  |  |
| **Oligonucleotides (5'-3')** |  |  |
|  |  |  |
| ***Oligonucleotides for amplification of CRP targets from bioinformatic screen*** | | |
| 13.73 For | GGCTGCGAATTCTTTTGTGATTAATTTCACAAAATAAGGTGTTATTCAGTGTGTGCTGCAATATTCAGGATGCATGA | This work |
| 13.73 Rev | GCCCGAAGCTTCATGTTATTCTCCATATCAGATAAATAGCACCTGTATTTCATGCATCCTGAATATTGCAGCACAC | This work |
| 13.77 For | GGCTGCGAATTCAATCCAATCATACTTTTTGTATGGTCGCTGGCGAAATAAAGTGATAAAAATCACATAA | This work |
| 13.77 Rev | GCCCGAAGCTTAAGTGATATGAAGGTTATATCCTTTTAATAAAAATTTTATGTGATTTTTATCACTTTA | This work |
| 12.57 For | GGCTGCGAATTCTATGATACACATCACAAAAAAATAAAAAAGTTTGCGCAATCGTTCTGATTTTGAT | This work |
| 12.57 Rev | GCCCGAAGCTTCATATTACCTCCGAAACACGTCGTCCACGAATATTTAAATCAAAATCAGAACGATTGC | This work |
| 11.10 For | GGCTGCGAATTCTTCGTACTGTCACCTTTTTAAATCATAAAAACTCTGATATTTGAACGAAGTCAAATTT | This work |
| 11.10 rev | GCCCGAAGCTTGAACGATTTTTTCCAAAAAATGATGATGAATAAAAGAAATTTGACTTCGTTCAAATAT | This work |
| 10.50 For | GGCTGCGAATTCTGAATCATATGCGATTCACCAATGAATCCTTTTTATAATGTAAAATAAATTAAAATA | This work |
| 10.50 Rev | GCCCGAAGCTTCATTAACTTATATTTTAAATAATGTATTTTAATTTATTTTACATTA | This work |
| 10.41 For | GGCTGCGAATTCCATGATGCAACTCACAAAAAAAATAAAAAAATTGCAAAATCCGTTTAACTAATCT | This work |
| 10.41 Rev | GCCCGAAGCTTCATGTTACCTCCCGTCATGTTGTTTCACGGATATTTGAGATTAGTTAAACGGATTTTG | This work |
| 9.86 For | GGCTGCGAATTCATATGTGATATTAATAGCACAAGTTCCTTACCAGATGAATTTGTAAAGAAC | This work |
| 9.86 Rev | GCCCGAAGCTTCATTCTTTGCCAGCAAATCCTGCACACGTGGGTTCTTTACAAATTCATCTG | This work |
| 8.89 For | GGCTGCGAATTCTTTTGTGGAGTGGGTTAAATTATTTACGGATAAAGTCACCAGAGGTGGAAAAATGAAA | This work |
| 8.89 Rev | GCCCGAAGCTTTTTCATTTTTCCACCTCTGGTGACTTTATCCGTAAATAATTTAACCCACTCCACAAAA | This work |
| 7.89 For | GGCTGCGAATTCTTTTCTCTGAAGCTCCCACTCCAGATGATGTAGTTCATTTTTTAACAACTTTATATTT | This work |
| 7.89 Rev | GCCCGAAGCTTCTTAAGAATAATATAGTAGAAAATCACAAAAAAAATATAAAGTTGTTAAAAAATAAAT | This work |
|  |  |  |
| ***Generation of different estA1 promoter fragments**** | | |
| *estA1 350 bp fragment For* | GGCTGCGAATTCATGAAAATAATATATAAAAAGCGAGTG | This work |
| *estA1 350 bp fragment Rev* | GCCCGAAGCTTTTAATAACATCCAGCACAGGCAGGATTAC | This work |
| PestA1 95 bp fragment For | GGCTGCGAATTCTAACATGATGCAACTCACAAAAAAAATAAAAAAATTGCAAAATCCGTTTAACTAATCT | This work |
| PestA1 95 bp fragment Rev | GCCCGAAGCTTCATGTTACCTCCCGTCATGTTGTTTCACGGATATTTGAGATTAGTTAAACGGATTTTG | This work |
|  |  |  |
| ***Generation of different estA2 promoter fragments**** | | |
| *estA2 460 bp fragment For* | GGCTGCGAATTCCCGGGGCGGTTCATTGTTATTTTTTTTGTG | This work |
| *estA2 460 bp fragment Rev* | GCCCGAAGCTTTTAATAGCACCCGGTACAAGCAGGATTACAAC | This work |
| PestA2 96 bp fragment For | GGCTGCGAATTCTAGTATGATACACATCACAAAAAAATAAAAAAGTTTGCGCAATCGTTCTGATTTTGAT | This work |
| PestA2 96 bp fragment Rev | GCCCGAAGCTTCATATTACCTCCGAAACACGTCGTCCACGAATATTTAAATCAAAATCAGAACGATTGC | This work |
| PestA2.1 93bp fragment For | GGCTGCGAATTCCATGATGCAACTCACAAAAAAATAAAAAAGTTTGCGCAATCGTTCTGATTTTGATTTAAAT | This work |
| PestA2.1 93bp fragment Rev | GCCCGAAGCTTCATATTACCTCCGAAACACGTCGTCCACGAATATTTAAATCAAAATCAGAACGATTGCGC | This work |
| PestA2.2 93bp fragment For | GGCTGCGAATTCTATGATACACATCACAAAAAAAATAAAAAAATTGCGCAATCGTTCTGATTTTGATTTAAAT | This work |
| PestA2.2 93bp fragment Rev | GCCCGAAGCTTCATATTACCTCCGAAACACGTCGTCCACGAATATTTAAATCAAAATCAGAACGATTGCGC | This work |
| PestA2.3 93bp fragment For | GGCTGCGAATTCTATGATACACATCACAAAAAAATAAAAAAGTTTGCAAAATCGTTCTGATTTTGATTTAAAT | This work |
| PestA2.3 93bp fragment Rev | GCCCGAAGCTTCATATTACCTCCGAAACACGTCGTCCACGAATATTTAAATCAAAATCAGAACGATT | This work |
| PestA2.4 93bp fragment For | GGCTGCGAATTCTATGATACACATCACAAAAAAATAAAAAAGTTTGCGCAATCCGTTTAACTAATCTTAAAT | This work |
| PestA2.4 93bp fragment Rev | GCCCGAAGCTTCATATTACCTCCGAAACACGTCGTCCACGAATATTTAAGATTAGTTAAACGGATT | This work |
| PestA2.5 93bp fragment For | GGCTGCGAATTCTATGATACACATCACAAAAAAATAAAAAAGTTTGCAAAATCCGTTTAACTAATCTTAAAT | This work |
| PestA2.5 93bp fragment Rev | GCCCGAAGCTTCATATTACCTCCGAAACACGTCGTCCACGAATATTTAAGATTAGTTAAACGGATTTTGCAA | This work |
| PestA2.6 93bp fragment For | GGCTGCGAATTCTATGATACACATCACAAAAAAATAAAAAAGTTTGCGCAATCGTTCTGATTTTGATTCAAAT | This work |
| PestA2.6 93bp fragment Rev | GCCCGAAGCTTCATATTACCTCCGAAACACGTCGTCCACGAATATTTGAATCAAAATCAGAACGATTGCGC | This work |
| PestA2.7 93bp fragment For | GGCTGCGAATTCTATGATACACATCACAAAAAAATAAAAAAGTTTGCGCAATCGTTCTGATTTTGATTTAAAT | This work |
| PestA2.7 93bp fragment Rev | GCCCGAAGCTTCATGTTACCTCCCGTCATGTTGTTTCACGGATATTTAAATCAAAATCAGAACGATTGCGC | This work |
|  |  |  |
| ***Generation of different eltAB promoter fragments**** | | |
| P*eltAB* 118 bp fragment For | GGCTGCGAATTCGCATGGATGTTTTATAAAAAACATGATTGACATCATGTTGCATATAGG | This work |
| P*eltAB* 118 bp fragment with CRP site mutated For | GGCTGCGAATTCGCATGGATGTTTTATAAAAAACAACATTGACATGTTGTTGCATATAGG | This work |
| P*eltAB* 118 bp fragment Rev | GCCCGAAGCTTCATCGAGGATATATATCATACAAGAAGACAATCCGGAAAAAGAT | This work |
| *eltAB 1127bp fragment For* | GGCTGCGAATTCTTCTGGTGTGGACTTTCTGGTGCTCCAGGTTGTG | This work |
| *eltAB 1127bp fragment Rev* | GCCCGAAGCTTTTCATTCCGAATCCTGTTATATATGTCAAC | This work |
|  |  |  |
| ***Primers used for amplification of different DNA targets in ChIP experiments*** | | |
| Psta2 For | GTTCTCGCAAGGACGAGAATTTC | This work |
| Psta2 Rev | GCCCGAAGCTTCATATTACCTCCGAAACACGTCGTCCACGAATATTTAAATCAAAATCAGAACGATTGC | This work |
| PeltAB For | GTTCTCGCAAGGACGAGAATTTC | This work |
| PeltAB Rev | GCCCGAAGCTTACAATCCGGAAAAAGATAACGCCAC | This work |
| *yabN For* | GGTGCGGCTGTCGAACAGTAAATAG | This work |
| *yabN Rev* | GCCGCGCGAATGGGAAACCCTCAG | This work |

*Note that the longest promoter fragment for each toxin was ordered from DNA2.0 rather than amplified by PCR.

**REFERENCES**

Crossman LC, Chaudhuri RR, Beatson SA, Wells TJ, Desvaux M, et al. (2010) A commensal gone bad: complete genome sequence of the prototypical enterotoxigenic *Escherichia coli* strain H10407. *J Bacteriol.* **192:**5822-5831.

Espert SM, Elsinghorst EA, Munson GP. (2011) The *tib* adherence locus of enterotoxigenic *Escherichia coli* is regulated by cyclic AMP receptor protein. *J Bacteriol*. **193:**1369-76.

Busby S, Kotlarz D, and Buc, H (1983) Deletion mutagenesis of the *Escherichia coli* galactose operon promoter region. *J. Mol. Biol*. **167:**259–274.

Lodge J, Fear J, Busby S, Gunasekaran P, Kamini NR. (1992) Broad host range plasmids carrying the *Escherichia coli* lactose and galactose operons. *FEMS Microbiol Lett*. **74:**271-6.

Kolb A, Kotlarz D, Kusano S, Ishihama A. (1995) Selectivity of the *Escherichia coli* RNA polymerase E sigma 38 for overlapping promoters and ability to support CRP activation. *Nucleic Acids Res.* **23:**819-26.
