# Supplementary material for: The Molecular Basis for Control of ETEC Enterotoxin Expression in Response to Environment and Host
Source: PLoS Pathog. 2015 Jan 8;11(1):e1004605. doi: 10.1371/journal.ppat.1004605 (PMC4287617; doi:10.1371/journal.ppat.1004605)
Supplement: S3 Table — All CRP binding sites on the ETEC H10407 chromosome identified by ChIP-seq. (DOCX) [file ppat.1004605.s007.docx]

**Table S3:** **All CRP binding sites on the ETEC H10407 chromosome identified by ChIP-seq**

| **Peak Centre^a^** | **Binding site(s)^b^** | **FAT^c^** | **Gene(s)d** | **K-12 Homologues^e^** |
| --- | --- | --- | --- | --- |
| 45284 | **TGTGA**TTGGTA**TCACA** | 1 | *ETEC_0040* | *caiT* |
| 56308 | n.d. | <1 | (ETEC_0052) | pdxA |
| 92014 | A**GTGA**TGGATG**TCAC**G | 1 | (*ETEC_0078*) | (*cra*) |
| 176905 | A**G**C**G**TTCCACG**TCACA** | <1 | *(ETEC_0150)* | *(hemL)* |
| 402526 | n.d. | <1 | (ETEC_0379)/ETEC_0380 | yahA/yahI |
| 408885 | **TGTGA**TCTCTC**TC**G**CA** | 1 | *ETEC_0385*/*ETEC_0386* | *yahN*/*yahO* |
| 461874 | **TGTG**CGCAAGA**TCACA** | <1 | *ETEC_0434* | *ddlA* |
| 463095 | TT**TG**CGCGAGG**TCACA** | <1 | *(ETEC_0436)* | *(phoA)* |
| 468009 | A**G**G**GA**TCTGCG**TCACA** | 10 | *ETEC_0443* | *aroM* |
| 491058 | n.d. | <1 | (ETEC_0462) | secF |
| 492973 | ATC**GA**TTGCGT**TCAC**G | 1 | *ETEC_0464* | ***tsx*** |
| 540805 | **TGTGA**TCTTTA**TCACA** | 1 | *ETEC_0511* | *maa* |
| 574230 | GA**TGA**CGACGA**TCACA** | <1 | *(ETEC_0538)* | *(ybaT)* |
| 586409 | n.d. | <1 | (ETEC_0549) | rhsD |
| 683187 | A**GTGA**TCGAGT**T**A**ACA** | 2 | *ETEC_0628* | ***cstA*** |
| 697540 | A**GTGA**TTTGCG**TCACA** | 2 | *ETEC_0639* | *rnk* |
| 739223 | C**GT**T**A**CCCTTG**TC**G**CA** | 4 | *ETEC_0680* | *rihA* |
| 941002 | **TGTGA**TGAGTA**TCAC**G | 2 | *ETEC_0869* | *ybiJ* |
| 958866 | **TGTGT**ACGAAA**TCACA** | <1 | *ETEC_0886/ETEC_0887* | ***ybiS/ybiT*** |
| 1128472 | n.d. | 1 | (*ETEC_1030*) | (*yccS*) |
| 1184239 | n.d. | <1 | ETEC_1083 | putA |
| 1205350 | A**GTGA**TGTAGA**TCACA**  **TGAGATCGAGCACACA** | 1 | *ETEC_1101* | *ycgZ* |
| 1263558 | **T**T**TGA**CGGCTA**TCAC**G | 1 | *ETEC_1166* | ***ptsG*** |
| 1274886 | **TGTGA**TCTGGA**TCACA** | 4 | *ETEC_1176*/*ETEC_1177* | ***ycfQ***/***bhsA*** |
| 1301786 | GA**TGA**TCCGCA**TCACA** | 1 | (*ETEC_1206*)/*ETEC_1207* | ETEC-specific/ETEC-specific |
| 1348166 | AT**TGA**ACAGGA**TCACA** | <1 | *(ETEC_1259)/ETEC_1260* | *(rluE)/icd* |
| 1376374 | G**GTGA**GCTGGC**TCACA** | 5 | *ETEC_1292*/*ETEC_1293* | ***ycgB***/***dadA*** |
| 1388620 | A**GTGA**GCCAGT**TAACA** | <1 | *(ETEC_1303)* | *(dhaL)* |
| 1541732 | CG**TGA**ACCGGG**TCACA** | <1 | *ETEC_1443/ETEC_1444* | *ycjZ/mppA* |
| 1567885 | GTTA**A**GTAAAA**TCACA** | <1 | *ETEC_1462/ETEC_1463* | ***paaZ/paaA*** |
| 1701402 | **TGTGA**TGGATG**TCACT** | <1 | *ETEC_1568* | *ydeN* |
| 1767726 | **TGTGA**TTAACAG**CACA** | 4 | *ETEC_1628* | ***mlc*** |
| 1777143 | **TGTGA**TCTAGCG**C**CA**A** | 1 | *ETEC_1637* | *pntA* |
| 1811426 | CG**TGA**TCAAGA**TCACG** | <1 | *(ETEC_1668A)* | (ETEC specific) |
| 1859265 | AT**TGA**GCGGGA**TCACA** | <1 | *(ETEC_1713)* | *(sufS)* |
| 1887513 | A**GTGA**TGCGCA**TCAC**G  TGCGAGGTGTGTCACA | 2 | *ETEC_1737* | *aroH* |
| 2126754 | **TGTG**GCGTGCA**TCACA** | 1 | n.a. | n.a. |
| 2201816 | G**GTGA**CGCGCG**TCACA** | 4 | *ETEC_2057* | *yedP* |
| 2210222 | C**GTGA**TCTCGCG**CACA** | 3 | *ETEC_2065*/*ETEC_2066* | *yedR*/ETEC-specific |
| 2458348 | **TGTGA**TCTGAA**TC**T**CA**  **TGCGATGCGTCGCGCA** | 1 | *ETEC_2278* | ***cdd*** |
| 2492757 | AT**TGA**TCGCCC**TCACA** | 7 | *ETEC_2309* | *yeiQ* |
| 2555083 | CG**TGA**CCAAAG**TCTCA** | <1 | *(ETEC_2360)* | *(yfaQ)* |
| 2729713 | TT**TGA**AGCTTG**TCACA** | 7 | *ETEC_2510*/*ETEC_2511* | *mntH*/*nupC* |
| 2735124 | A**GT**T**A**TTCATG**TCAC**G | 1 | *ETEC_2514* | *yfeC* |
| 2795423 | **TGTGA**GCCATGA**CACA** | <1 | *(ETEC_2572)/ETEC_2573* | ***(aegA)/narQ*** |
| 2810983 | C**GTGA**TCAAGA**TCACA** | 6 | *ETEC_2586* | ***hyfA*** |
| 2867803 | n.d. | <1 | tRNA-Arg/ETEC_2638 | ETEC specific |
| 2887131 | **T**T**TGA**TCTCGC**TCACA** | 10 | (*ETEC_2666*)/*ETEC_2665* | (***xseA***)/***guaB*** |
| 3012645 | **TGTGA**TCCCCACA**ACA** | 1 | (*ETEC_2793*) | (***ung***) |
| 3048307 | TT**TGA**CGAGCA**TCACC** | <1 | *(ETEC_2822)* | *(emrB)* |
| 3132920 | G**GTGA**CCGGTT**TCACA** | 6 | *ETEC_2905*/*ETEC_2906* | *ascG*/*ascF* |
| 3161660 | **TGTGA**CCGTGG**TC**G**CA** | 3 | (*ETEC_2933*) | (***nlpD***) |
| 3184337 | CG**TGA**TGCGTG**T**A**ACA** | <1 | *(ETEC_2956)/ETEC_2955* | *(cysI)/cysH* |
| 3196088 | **TGTGA**TTACGA**TCACA** | 1 | *ETEC_2966*/*ETEC_2967* | *ygcW*/*yqcE* |
| 3223792 | A**GTGA**TCTTGA**TC**T**CA**  **AGTTATGTATCTATCA** | 1 | *ETEC_2986* | *sdaC* |
| 3234980 | **TG**C**GA**TCGTTA**TCACA** | 1 | (*ETEC_2994*)/*ETEC_2995* | (*fucU*)/*fucR* |
| 3265047 | **TGTGA**CCTGGG**TCAC**G | 7 | *ETEC_3017* | *rppH* |
| 3324543 | **TGTG**GGCTACG**T**A**ACA** | <1 | *(ETEC_3075)* | *(ydhD)* |
| 3361162 | n.d. | 1 | *ETEC_3105* | ***serA*** |
| 3368992 | **T**T**TGA**TGCACCG**CACA** | 1 | (*ETEC_3113*) | (*ygfI*) |
| 3382158 | **TGTGA**TCTACAA**CAC**G | 16 | *ETEC_3126* | *cmtB* |
| 3390811 | **TGTGA**TTTGCT**TCACA** | 4 | *ETEC_3133* | ***galP*** |
| 3408173 | **TGTGA**TGTGGA**T**A**ACA** | 1 | *ETEC_3154* | ***nupG*** |
| 3442697 | **TGTGA**TGATTG**TC**G**CA** | 1 | *ETEC_3186* | ETEC-specific |
| 3558573 | A**GTGA**TTTGGC**TCACA** | 4 | *ETEC_3291* | *ygiS* |
| 3580767 | A**GTGA**CTTGCA**TCACA** | 2 | (*ETEC_3318*) | (*yqiH*) |
| 3635301 | AT**TGA**TCTAAC**TCAC**G | 1 | *ETEC_3362* | ***uxaC*** |
| 3635965 | n.d. | <1 | (ETEC_3363) | **exuT** |
| 3642302 | CT**TGA**AGTGGG**TCACA** | <1 | *(ETEC_3372)* | *(yqjG)* |
| 3665634 | **TGTGA**TCAATG**TCA**AT  TGTGCTTTAGCGCGCA | 1 | *ETEC_3393*/*ETEC_3394* | *garP*/*garD* |
| 3673865 | n.d. | <1 | (ETEC_3402) | agaA |
| 3675849 | n.d. | <1 | (ETEC_3403)/ETEC_3404 | agaS/kbaY |
| 3682270 | n.d. | <1 | (ETEC_3411) | yraL |
| 3721308 | G**GTGA**TTGATG**TCAC**C | 1 | (*ETEC_3446*) | (*greA*) |
| 3759898 | n.d. | <1 | ETEC_3486 | **nanA** |
| 3785700 | CG**TG**GGTCGCA**TCACA** | <1 | *(ETEC_3510)* | *(mreC)* |
| 3878729 | G**GTGA**TTTTGA**TCAC**G | 20 | *ETEC_3614*/*ETEC_3615* | *ppiA*/*tsgA* |
| 3908574 | G**GTGA**TCGCGC**TCACA** | 2 | (*ETEC_3645*) | (***hofM***) |
| 3910075 | n.d. | <1 | (ETEC_3646) | **mrcA** |
| 3918861 | **TGTGA**GTGGAA**TC**G**CA** | <1 | *ETEC_3652/ETEC_3653* | *yhgE/pck* |
| 3928891 | n.d. | <1 | (ETEC_3659) | feoB |
| 3986400 | C**GTGA**TTTTATC**CACA** | 2 | *ETEC_3707* | ***rpoH*** |
| 4000452 | n.d. | <1 | (ETEC_3722)/ETEC_3723 | nikA/nikB |
| 4105040 | AG**T**A**A**GGCAAG**TC**C**C**T | <1 | n.a. | *n.a.* |
| 4111116 | **TGTGA**CGGGGC**T**A**ACA** | 1 | (*ETEC_3806*) | (*wecH*) |
| 4153055 | **TGTGA**TCTGAA**TCACA**  **TGTGATCTACAGCATG** | 45 | *ETEC_3840* | ***yibI*** |
| 4153191 | **TGTGA**TTGATA**TCACA**  **TGTGATGAACGTCACG** | 27 | *ETEC_3841* | ***mtlA*** |
| 4158433 | n.d. | 1 | *ETEC_3846* | *lldP* |
| 4159392 | n.d. | <1 | (ETEC_3846) | lldp |
| 4196869 | **TG**CA**A**TCGATA**TCACA** | <1 | *ETEC_3886* | *dinD* |
| 4205722 | n.d. | <1 | (ETEC_3893) | recG |
| 4210231 | n.d. | <1 | (ETEC_3896) | yicH |
| 4242727 | n.d. | <1 | (ETEC_3922)/ETEC_3921 | ETEC specific |
| 4251326 | CT**T**ACTCCTGC**TCACA** | <1 | *ETEC_3938* | ETEC specific |
| 4266125 | G**GTGA**TGGCATC**C**G**C**G | 4 | (*ETEC_3956*) | (*nepI*) |
| 4290730 | GG**TGA**GCAAAAC**CAC**G | <1 | *(ETEC_3979)* | *(yidR)* |
| 4296631 | n.d. | <1 | (ETEC_3985)/ETEC_3984 | dgoR/dgoK |
| 4305618 | n.d. | <1 | (ETEC_3992) | dnaA |
| 4309544 | n.d. | <1 | (ETEC_3997) | trmE |
| 4322430 | AT**TGA**CCTGAG**TCACA** | <1 | *(ETEC_4010)* | *(yieL)* |
| 4340544 | CT**TGA**CCACGG**TCA**G**A** | <1 | *(ETEC_4025)/ETEC_4024* | *(atpA)/atpG* |
| 4344399 | n.d. | <1 | (ETEC_4030)/ETEC_4029 | atpI/atpB |
| 4344649 | **TGTGA**TCTGAAG**CAC**G | 2 | *ETEC_4030* | *atpI* |
| 4347566 | n.d. | <1 | (ETEC_4032) | gidA |
| 4358905 | n.d. | <1 | (ETEC_4042) | rbsB |
| 4373517 | **TGT**A**A**TGCTGG**T**A**ACA** | <1 | *(ETEC_4051)* | *(ilvG)* |
| 4386425 | n.d. | <1 | (ETEC_4062)/ETEC_4061 | **rhlB/gppA** |
| 4393016 | n.d. | <1 | (ETEC_4068)/ETEC_4069 | rffE/rffD |
| 4402013 | C**GTG**CTGCATA**TCAC**G | <1 | *(ETEC_4077)* | *(rffM)* |
| 4404357 | n.d. | <1 | (tRNA-Arg)/tRNA-His |  |
| 4408568 | n.d. | <1 | n.a. |  |
| 4409337 | n.d. | <1 | (ETEC_4081) | hemY |
| 4409915 | n.d. | <1 | (ETEC_4082)/ETEC_4081 | hemX/hemY |
| 4412999 | C**GTGA**TCAATT**T**A**ACA** | 2 | *ETEC_4085*/*ETEC_4085* | *hemC*/*cyaA* |
| 4431648 | n.d. | <1 | (ETEC_4102) | pldB |
| 4436859 | n.d. | <1 | (ETEC_4106) | metE |
| 4437695 | n.d. | <1 | (ETEC_4107) | **ysgA** |
| 4438352 | G**GTGA**TGAGTA**TCAC**G  TGTGATTTGAATCACT | 4 | *ETEC_4107*/*ETEC_4108* | *ysgA*/*udp* |
| 4508745 | **TGTGA**TATTTG**TCACA** | 20 | (*ETEC_4165*)/*ETEC_4164* | (*fdhD*)/*fdoG* |
| 4517442 | C**GTGA**TCGCTG**TC**C**CA** | 1 | (*ETEC_4173*) | (*rhaA*) |
| 4531273 | n.d. | <1 | (ETEC_4186) | sbp |
| 4564670 | **TG**C**GA**TCCGCC**TCA**T**A** | 1 | *ETEC_4216*/*ETEC_4217* | *ptsA*/*frwC* |
| 4601910 | n.d. | <1 | n.a. |  |
| 4615956 | n.d. | <1 | (ETEC_4253)/ETEC_4252 | thiE/thiF |
| 4668870 | **TGT**A**A**CAGAGA**TCACA** | <1 | *ETEC_4289/ETEC_4290* | ***malE/malK*** |
| 4725047 | **TGTG**CGGATGA**TCACA** | <1 | n.a. | n.a. |
| 4731402 | **TGTGA**TCTTGCG**CA**T**A** | 3 | (*ETEC_4365*) | (*aphA*) |
| 4737089 | n.d. | <1 | ETEC_4370 | yjcB |
| 4761367 | C**GTGA**TGGCTG**TCAC**G | 1 | *ETEC_4389* | *fdhF* |
| 4793507 | n.d. | <1 | (ETEC_4419) | **proP** |
| 4812882 | n.d. | <1 | (ETEC_4433) | **dcuB** |
| 4819937 | n.d. | <1 | (ETEC_4441) | yjdL |
| 4846352 | n.d. | 1 | *ETEC_4464* | ETEC-specific |
| 4848117 | C**GTGA**GTTCTG**TCACA** | 3 | n.a. | n.a. |
| 4863253 | **T**T**TGA**TCAACA**TC**G**CA** | 1 | (*ETEC_4478*) | (ETEC-specific) |
| 4873926 | G**GTGA**TCTATT**TCACA** | 3 | *ETEC_4486*/*ETEC_4487* | *aspA*/*fxsA* |
| 4930149 | **TGTGA**TGAACT**TCA**A**A** | 1 | *ETEC_4545*/*ETEC_4546* | *yjfY*/*rpsF* |
| 4940903 | **TGTGA**TCACTA**TC**G**CA** | 4 | *ETEC_4557*/*ETEC_4558* | ETEC-specific/*ytfA* |
| 4993073 | **TGTGA**CTGGTA**TC**T**C**G | 1 | (*ETEC_4604*) | (*valS*) |
| 5002854 | **TGT**A**A**CCTTTG**TCACA** | <1 | *ETEC_4610/tRNA-Leu* | *yjgB/tRNA-Leu* |
| 5030724 | **TG**C**GA**TGAATG**TCACA** | 1 | *ETEC_4633*/*ETEC_4634* | *gntP*/*uxuA* |
| 5129400 | CG**T**ACCGTCGG**TCACA** | <1 | *(ETEC_4736)* | *(yjjI)* |
| 5129944 | **TGTGA**TGTATA**TC**GA**A** | 15 | *ETEC_4736*/*ETEC_4737* | *yjjI*/*deoC* |

^a^ Genome coordinate of the ChIP-seq peak in H10407. Underlined text indicates that the ChIP-seq peak maps to sequence that is not conserved in *E. coli* K-12.

^b^ CRP binding site sequence predicted by MEME. Bold text indicates a match to the known CRP consensus site (TGTGAn_6_TCACA). In some cases two sites were found. “n.d.” indicates that MEME did not detect a putative binding site.

^c^ “Fold Above Threshold” score indicating the level of CRP association as determined by ChIP-seq. Note that the FAT score refers to the high stringency cut off for binding site selection. Hence, those sites scoring <1, passed only the low stringency cut-off.

^d^ Genes in parentheses indicate that the ChIP-seq peak is located within that gene. Downstream genes are only listed if the annotated gene start is ≤ 300 bp downstream of the CRP ChIP-seq peak. “n.a.” indicates that no genes starts are ≤ 300 bp from the CRP ChIP-seq peak.

^e^ *E. coli* K-12 homologues are listed for the ETEC genes in the previous column. Genes in parentheses indicate that the ChIP-seq peak is located within that gene. “n.a.” indicates that no genes starts are ≤ 300 bp from the CRP ChIP-seq peak. “ETEC-specific” indicates that there is no K-12 homologue. Underlined genes have been identified as CRP targets in a previous ChIP-chip study (Grainger *et al*., 2005). Bold genes are listed as CRP targets in the Ecocyc database.
